# Assessment of validity, reliability, responsiveness and acceptability of seven Dutch-Flemish PROMIS computerised adaptive tests (CATs) in Dutch people with type 2 diabetes: an observational and qualitative study

**DOI:** 10.1136/bmjopen-2024-087898

**Published:** 2025-11-28

**Authors:** Lenka Groeneveld, Caroline B Terwee, Esmee M van der Willik, Frans J van Ittersum, Marlous Langendoen-Gort, Fleur Pals, Marieke T Blom, Joline W J Beulens, Petra J M Elders, Femke Rutters

**Affiliations:** 1 Epidemiology & Data Science, Amsterdam UMC, Amsterdam, Netherlands; 2 Amsterdam Public Health research institute, Health Behaviors & Chronic Diseases, Amsterdam UMC, Amsterdam, Netherlands; 3 Amsterdam Public Health research institute, Methodology, Amsterdam UMC, Amsterdam, Netherlands; 4 Department of Clinical Epidemiology, Leiden University Medical Center, Leiden, Netherlands; 5 Department of Nephrology, Amsterdam University Medical Center, Amsterdam, Netherlands; 6 Department of General Practice & Elderly Care Medicine, Amsterdam UMC Location VUmc, Amsterdam, Netherlands; 7 Amsterdam Cardiovascular Sciences, Amsterdam UMC, Amsterdam, Netherlands; 8 Julius Center for Health Sciences and Primary Care, University Medical Center Utrecht, Utrecht, Netherlands

**Keywords:** General diabetes, Patient Reported Outcome Measures, Diabetes Mellitus, Type 2

## Abstract

**Abstract:**

**Objectives:**

This study aimed to assess construct validity against commonly used patient-reported outcome measures (PROMs), test–retest reliability and responsiveness of seven Dutch-Flemish Patient-Reported Outcomes Measurement Information System (PROMIS) computerised adaptive testing (CATs) in Dutch adults with type 2 diabetes (T2D), and assess their acceptability in healthcare providers and people with T2D.

**Design:**

A cross-sectional observational study in people with T2D and qualitative study involving both people with T2D and healthcare professionals.

**Setting:**

Participants with T2D were recruited from the ongoing Hoorn Diabetes Care System cohort in the West-Friesland area of the Netherlands. Additionally, people with T2D and advanced chronic kidney disease were recruited at the outpatient clinics of Amsterdam University Medical Centre and ‘Niercentrum aan de Amstel’, both in the Amsterdam area of the Netherlands. The healthcare professionals involved in the qualitative part were recruited at the Amsterdam University Medical Centre.

**Participants:**

314 people with T2D (age 64.0±10.8 years, 63.7% men).

**Primary and secondary outcome measures:**

Participants completed seven PROMIS CATs (assessing (1) Physical Function, (2) Pain Interference, (3) Fatigue, (4) Sleep Disturbance, (5) Anxiety, (6) Depression and (7) Ability to Participate in Social Roles and Activities), and PROMs measuring similar constructs. After 2 weeks and 6 months, participants completed the CATs measures again, together with seven Global Rating Scales (GRS) on perceived change in each domain. Construct validity was assessed using Pearson’s correlations. Test–retest reliability was assessed by the intraclass correlation coefficient (ICC). Measurement error was assessed by the standard error of measurement (SEM) and minimal detectable change (MDC). Responsiveness was assessed by correlations between change scores on the PROMIS CAT and GRS. Acceptability was assessed through focus groups and interviews in healthcare providers and people with T2D.

**Results:**

Except for Fatigue, all PROMIS CAT domains demonstrated sufficient construct validity, since ≥75% of the results was in accordance with a priori hypotheses. All seven PROMIS CATs showed sufficient test–retest reliability (ICCs 0.73–0.91). SEM and MDC ranged from 2.1 to 2.7 and from 5.7 to 7.4, respectively. Responsiveness was rated as insufficient in this study design as there was almost no change in participants’ own rating of their health compared with 6 months ago according to a global rating of change.

During the focus groups and interviews, healthcare providers and people with T2D agreed that CATs could serve as a conversation starter in routine care, but should never replace personal consultations with a doctor. If implemented, participants would be willing to spend 15 min to complete the PROMIS CATs.

**Conclusions:**

The PROMIS CATs showed sufficient construct validity and test–retest reliability in most domains in people with T2D. Responsiveness needs to be evaluated in a population with poorer diabetes control or in a study design with longer follow-up. The CATs are well accepted to be used in care to identify relevant topics, but should not replace personal contact with the doctor.

**STRENGTHS AND LIMITATIONS OF THIS STUDY**

- A strength is that we used a high-quality study design and large sample size.
- A second strength is that we used an observational and qualitative study design, involving both people with type 2 diabetes as well as healthcare professionals.
- A limitation is the relatively short duration of this study and our population having good glycaemic control, resulting in no change in self-reported health in our population, while change is a requirement for assessing responsiveness.
- A second limitation is that, although offered an alternative way to participate, participants who did not have access to an electronic device with internet connection did not participate.
- A third limitation is that the focus groups were perhaps too small to draw strong conclusions.

## Introduction

 The nationally and internationally recommended Patient-Reported Outcomes Measurement Information System (PROMIS) is a collection of patient-reported outcome measures (PROMs), used to measure a variety of health-related quality of life (HRQOL) domains.^1^,^3^ These PROMs capture several domains that are commonly relevant across diseases. For each domain, different types of PROMs were developed. The basis consists of an item bank for each domain, containing a large number of questions. From this item bank, short forms of four to eight items were developed. In addition, the item bank can be used for computerised adaptive testing (CAT), an efficient administration method in which the computer selects questions from an item bank based on the answers to previous questions, until a predefined reliability or a maximum number of items is administered. With CATs, PROMs are adapted to the symptom severity or functional level of the patient, generally resulting in less items and more relevant questions.^4^ The psychometric properties as well as feasibility of using PROMIS CATs have recently been evaluated in other populations, such as rehabilitation patients,^5^ people with stroke^6^ and people with advanced chronic kidney disease (CKD).^7^ PROMIS CATs have been shown to have better measurement properties than traditional generic PROMs and similar ones as disease-specific PROMs, making PROMIS CATs suitable for use in clinical practice.^8^,^10^

In our systematic review on PROMs for people with type 2 diabetes (T2D), we showed that a large number of PROMs that intend to measure (aspects of) HRQOL are available.^11^,^14^ However, the PROMs currently used in the field of T2D have limitations: the PROMs measure a large variety of (sub)constructs, which are not all HRQOL constructs, with a small number of PROMs not measuring HRQOL at all. There is a need for consensus on which aspects of HRQOL should be measured in people with T2D and which PROMs to use in research and daily practice. The PROMIS CATs could provide that solution.

Currently, several Dutch-Flemish PROMIS CATs are available for use in the Netherlands. Seven of those CATs assess commonly relevant general HRQoL domains: (1) Physical Function, (2) Pain Interference, (3) Fatigue, (4) Sleep Disturbance, (5) Anxiety, (6) Depression and (7) Ability to Participate in Social Roles and Activities. At the moment, no T2D-specific domains, such as diabetes distress, are available yet as CATs. The domains that are available showed sufficient construct validity against commonly used PROMs and test–retest reliability in Dutch patients with advanced CKD.^7^ However, we do not know the validity, reliability, responsiveness and acceptability of these CATs in Dutch people with T2D and whether and how they could be implemented in routine care, since other HRQoL issues might be important for people with T2D, that is, higher risk of depression due to certain medication use or impairment in physical function due to neuropathy. This study, therefore, aimed to assess construct validity against commonly used PROMs, test–retest reliability and responsiveness of the above-mentioned seven Dutch-Flemish PROMIS CATs in Dutch people with T2D, and assess their acceptability in healthcare providers and people with T2D.

## Methods

### Study design and population

This study has a two-stage design. First, we assessed construct validity, test–retest reliability and responsiveness of seven PROMIS CATs in a large group of people with T2D, measuring at baseline, after 2 weeks and 6 months. Second, we conducted two interviews and two focus groups with healthcare providers and people with T2D to assess acceptability, by determining which PROMIS domains are most relevant for T2D care and how to implement the validated CATs in clinical practice and feedback their results to doctors and patients.

**Figure 1:**
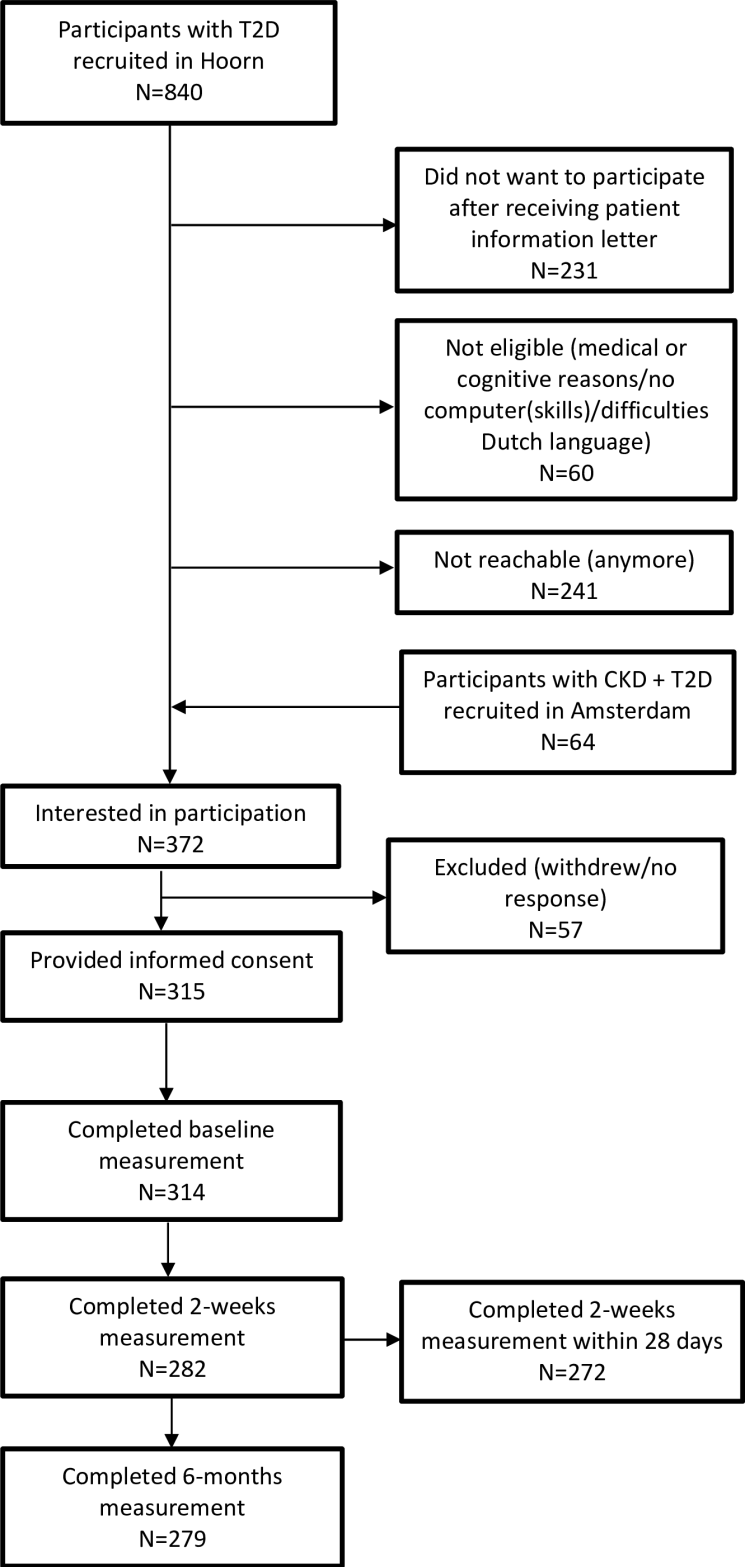
Flow chart of participant inclusion for baseline, 2 weeks and 6 months measurements. CKD, chronic kidney disease; T2D, type 2 diabetes.

The first stage of the study was conducted in parallel and using the same methods as our colleagues who aimed to assess the psychometric properties of CATs in patients with advanced CKD, as described in detail in van der Willik *et al*^7^ and Terwee *et al*.^15^ For stage 1, participants were eligible if they were older than 18 years of age, had T2D, a good command of the Dutch language, and if they were able to provide consent for participation. Participants were excluded from this study if they were unable to complete the questionnaires due to cognitive impairment. Participants were recruited from the ongoing Hoorn Diabetes Care System cohort, which is a prospective cohort of almost all people with T2D in the West-Friesland area of the Netherlands.^16^ This study was performed in a random subgroup of people who have given permission to be contacted for additional research. Additionally, people with T2D and advanced CKD were recruited at the outpatient clinics of Amsterdam University Medical Centre and ‘Niercentrum aan de Amstel’, both in the Amsterdam area of the Netherlands.^15^ A total of 840 potential participants were contacted, of which 372 were interested in receiving the patient information letter. After receiving the letter, 315 participants provided informed consent (figure 1). For stage 2 of the study, for patient participants, the same inclusion criteria applied as in part one. The healthcare professionals working in different specialties and at different levels (ie,doctor, diabetes nurse) were recruited within the Amsterdam University Medical Centre or University Medical Centre Utrecht.

For both parts of the study, eligible participants received written information by email and were, if needed, approached by telephone after 2 weeks for further information. After providing written informed consent for stage one, participants were invited by email to complete the CATs and other PROMs digitally at the KLIK research platform (www.hetklikt.nu)^17^ at three time points; at inclusion (ie, baseline), after 2 weeks and after 6 months. If necessary, two reminders to complete the PROMs were sent by email or participants were contacted by telephone. During follow-up, participants received care as usual, which could include changes in medication. The study was designed and reported according to COnsensus-based Standards for the selection of health Measurement Instruments (COSMIN) guidelines. The COSMIN initiative aims to improve the selection of outcome measurement instruments of health outcomes by developing and encouraging the use of transparent methodology and practical tools for selecting the most suitable outcome measurement instrument in research and clinical practice. COSMIN developed design requirements and preferred statistical methods of studies on measurement properties in an international Delphi study.^18^ Based on these guidelines, we aimed to include 300 participants. According to COSMIN, a sample size of 100 was considered ‘very good’ for construct validity, test–retest reliability and responsiveness, so we more than complied with the COSMIN guidelines.^19^ For stage 2, participants joined one of the interviews or focus groups, which were conducted live or via Zoom and led by one of the senior researchers experienced in qualitative research.

### Patient and public involvement

Patients or the public were not involved in the design, conduct, reporting or dissemination plans of this study.

### Measures

#### Stage 1: assessment of validity, reliability and responsiveness

We collected the following information from the medical records of the participants, if available: age, sex, educational level, ethnicity, diabetes duration, smoking status, haemoglobin A1c (HbA1c) levels, fasting glucose levels, total cholesterol levels, low-density lipoprotein and high-density lipoprotein cholesterol levels, triglyceride levels, body mass index (weight (kg)/height (m)^2^), systolic and diastolic blood pressure and glomerular filtration rate (eGFR) estimated according to the Modification of Diet in Renal Disease formula.^20^

Participants completed the following PROMs on the KLIK research platform,^17^ which is connected to the CAT software of the Dutch-Flemish Assessment Center, part of the Dutch-Flemish PROMIS National Center^21^:

Seven Dutch-Flemish PROMIS CATs^22^:

- PROMIS CAT Physical Function v1.2
- PROMIS CAT Pain Interference v1.1
- PROMIS CAT Fatigue v1.0
- PROMIS CAT Sleep Disturbance v1.0
- PROMIS CAT Anxiety v1.0
- PROMIS CAT Depression v1.0
- PROMIS CAT Ability to Participate in Social Roles and Activities v2.0.

All items of these CATs have five response options (eg, ranging from ‘never’ to ‘always’ or from ‘not at all’ to ‘very much’). In this study, the CAT stopped when an SE of 2.2 on the T-score metric was reached (comparable to a reliability of approximately 0.95) or when a maximum of 12 items per CAT was administered. We used a lower SE compared with the standard stopping rule (ie, SE: 3.0),^4^ because a higher reliability may be preferable for routine care and by using this setting, the optimal performance of PROMIS CATs could be investigated. PROMIS CAT scores were calculated based on the original US item parameters, as per PROMIS convention, and were expressed as T-scores where a score of 50 represents the average score of the US general population, with an SD of 10. Higher scores indicate more of the construct (eg, a higher score for Depression means more depression, a higher score for Physical Function means more (better) function).

In addition, we used the following regular PROMs (not CATs) to assess construct validity of the seven PROMIS CATs:

- The Short-Form Health Survey 12 (SF-12) version two is a generic PROM consisting of twelve items assessing the following eight aspects of HRQOL: physical functioning, role-physical, bodily pain, general health, vitality, social functioning, role-emotional and mental health.^23^ A physical component summary (PCS) score and a mental component summary score were calculated based on weighted summaries of all items, using the standard SF-12 scoring algorithm. To enable comparison with PROMIS domains, we also calculated eight domain scores (not part of the official SF-12 scoring). For physical functioning, physical and emotional role functioning, and mental health, the two available items were summarised. For bodily pain, vitality, social functioning and general health, single items were used. Domain scores were transformed to a score from 0 to 100, while the PCS and PCS scores have a mean of 50, representing the average score of the US general population, with an SD of 10. Higher scores indicate a better HRQOL.
- The audit of diabetes-dependent quality of life is a PROM to assess the impact of diabetes on overall quality of life,^24 25^ which was translated to Dutch and validated. In this study, only the two general quality of life questions and the domain Leisure Activities, which consists of an impact and an importance question, were included. The impact question asks how participants would enjoy their leisure activities if they did not have diabetes. This question consists of five response options, ranging from −3 (‘a lot more’) to 1 (‘less’). The importance question asks how important leisure activities are to the participants and consists of four response options, ranging from 0 (‘very important’) to 3 (‘not at all important’). A weighted impact score can then be calculated by multiplying the impact rating and importance rating, ranging from −9 (maximum negative impact of diabetes) to 3 (maximum positive impact of diabetes). Higher scores indicate better quality of life.
- The Patient Health Questionnaire-9 (PHQ-9) is a PROM consisting of nine items to assess the severity of depressive symptoms according to the Diagnostic and Statistical Manual of Mental Disorders (DSM)-5 criteria,^26 27^ which was translated to Dutch and validated. Every question consists of four response options ranging from ‘not at all’ to ‘almost every day’. A maximum of three points can be scored for each question and the maximum number of points is 27. The higher the score, the greater the indication of depressive symptoms.
- The EuroQol five dimensions (EQ-5D) consists of five questions to assess HRQOL in the following five dimensions: mobility, self-care, usual activities, pain/discomfort and anxiety/depression,^28 29^ which was translated to Dutch and validated. In this study, the three-level EQ-5D version (EQ-5D-3L) was used. Every question consists of three response options: ‘no problem’, ‘some problem’, ‘extreme problem/unable to’ (labelled 1–3). In addition, participants were asked to compare their health on the day of completing the questionnaire with their general health 12 months ago, with three response options (‘better’, ‘same’ or ‘worse’). The EQ-5D-3L index is calculated by subtracting the values of the descriptive EQ-5D system from the numerical value 1. This corresponds to the best possible health status, while an index value of <0 represents the worst possible health status.
- The Problem Areas in Diabetes Scale 20 is a PROM consisting of 20 questions to assess diabetes-related distress,^30 31^ which was translated to Dutch and validated. Each question consists of five response options ranging from ‘not a problem’ to ‘serious problem’. The maximum number of points that can be scored per question is four. This results in a total score from 0 to 80 points. A higher score indicates more diabetes-related distress.
- The PROMIS v1.2 Global Health Scale consists of 10 items, representing a total of 5 health domains (pain, fatigue, physical function, emotional distress and social health) as well as general health, cutting across these domains. Two subscale scores were calculated, namely Global Mental Health (GMH) and Global Physical Health (GHP), each consisting of four items. Each item is scored on a 5-point scale, except for question Global07r, which is scored on an 11-point scale and then recoded to a 5-point scale. GMH and GHP scores are expressed as T-scores where a score of 50 represents the average score of the US general population, with an SD of 10. A higher score indicates better global mental and/or physical health.^32^

Additionally, participants completed the PROMIS item v1.0 Numerical Rating Scale Pain Intensity 1a, which is a single item with a 0–10 scale, with higher scores indicating more pain (0 meaning no pain and 10 meaning worst pain imaginable).

At 6 months follow-up, participants were asked to rate their perceived change in each of the seven PROMIS domains on a Global Rating Scale (GRS) (eg,‘How did your fatigue change compared with 6 months ago?’). Perceived change was rated on a 5-point scale (much improved, a little improved, not changed, a little deteriorated, much deteriorated).

Only the seven PROMIS CATs and the PROMIS item v1.0 Numerical Rating Scale Pain Intensity 1a were completed at all three time points, the other PROMs were completed at baseline only. The PROMs were presented in a fixed order (first the PROMIS CATs and PROMIS Pain Intensity single item, then the other PROMs) both within as well as across participants, but the items asked could differ within participants during follow-up and across participants, depending on their answers. The KLIK platform did not allow for any missing values within questionnaires, except when participants would stop completing questionnaires.

#### Stage 2: acceptability

The focus groups were led by one of the senior scientists, while the interviews were conducted by the junior researcher, both with experience in qualitative research. The focus groups took place at the end of October 2021, after participants had completed the baseline, 2 weeks and 6 months measurements. Each focus group and interview took about 1 hour and was audio recorded. During the focus groups and interviews, a standardised script including predefined questions was followed. For the full list of questions, please see online supplemental table 1. In short, first the different PROMIS domains were introduced and it was then assessed whether the domains were considered relevant, for example by asking the participants: “Are these domains the most relevant to assess the impact of diabetes? And could you rate them, one being most important, seven least important?”. Furthermore, we assessed whether the whole set was considered comprehensive for the measurement of self-rated health. In the second part of the focus group, using the principles of the Extended Normalisation Process Theory (ENPT), we discussed how to implement the CATs in clinical practice and how we should feedback their results to doctors and people with T2D, by asking “What are the factors in current practice that facilitate measuring PROs in regular care for people with diabetes? Could this be achieved by that is, Tablet-PCs or laptops, extra time consultation?”. ENPT conceptualises the sociostructural and social-cognitive resources that people draw on to realise an implementation and offers a useful framework for explaining implementation processes and the role played by intervention context interactions. ENPT has four main constructs, namely potential (concerns, change valence and change efficacy), capability (workability and integration), capacity (structure, resources, norms and roles) and contribution (for which potential, capability and capacity form the context). The discussion was focused on what the participants identified as chances and options, but also risk and limitations. All questions were open-ended and questions were posed until no new categories, themes or explanations emerged from the participants. In addition, to thoroughly investigate all expressions of the participants, the moderator asked in-depth questions such as: ‘In what way do you mean that or could you provide us with an example?’.

### (Statistical) analyses

#### Stage 1: assessment of validity, reliability and responsiveness

Baseline characteristics were expressed as a percentage for dichotomous variables, as means and SD for normally distributed continuous variables, and as medians and IQRs for non-normally distributed variables. Percentages of missing variables varied between 15.9% for baseline eGFR and 52.2% for educational level. Data analysis was performed using SPSS V.28.0 (SPSS).

### Construct validity

Pearson’s correlations were used to assess construct validity of the PROMIS CATs. A priori we formulated hypotheses on the expected correlations between each PROMIS CAT domain and the other PROMs based on literature and expert judgement. Regarding convergent validity, we hypothesised strong correlations (r≥0.7) between PROMIS CATs and comparable PROM domains and moderate correlations (r=0.5–0.7) between PROMIS CATs and largely related PROM domains. Regarding divergent validity, we hypothesised weaker correlations (r≤0.) for other comparisons (online supplemental table 2). If ≥75% of the results was in accordance with the hypotheses, construct validity was considered sufficient.

### Test–retest reliability

Two aspects of test–retest reliability were assessed: reliability (defined as the proportion of the total variance in the measurements which is due to true differences between patients) and measurement error (defined as the systematic and random error of a patient’s score that is not attributed to true changes in the construct to be measured).^19^

To assess reliability, the intraclass correlation coefficient (ICC) was calculated in participants with a valid baseline and 2-week measurement. A measurement is considered valid when both measurements were completed by the participants within a maximum of 28 days. The ICC was calculated using a two-way random-effects model for absolute agreement: $ICCagreement=\frac{\sigma_{p}^{2}}{\sigma_{p}^{2}+\sigma_{m}^{2}+\sigma_{e}^{2}}$,

whereby $\sigma_{p}^{2}$ is the variation between patients, $\sigma_{m}^{2}$is the variation between measurements and $\sigma_{e}^{2}$ is the random error variance. The ICC was computed for each CAT domain as well as PROMIS Pain Intensity single item separately. We considered an ICC ≥0.70 as sufficient.

To assess measurement error, the standard error of measurement (SEM) was calculated for each CAT domain separately. SEM was calculated by dividing the SD of the difference between test and retest scores (SDdiff) by √2. A mean SEM of each domain was subsequently calculated for the whole group. The SEM is expressed in the unit of measurement of the PROM scale and lower scores mean less measurement error.

The minimal detectable change (MDC) was calculated to facilitate interpretation of PROMIS change scores in clinical practice. The MDC was calculated for each CAT domain separately. The MDC is defined as the smallest change in score that can be detected beyond measurement error, with 95% CI. Being based on item response theory (IRT), PROMIS CATs, the MDC differs per participant (because with IRT the SE of each score is different) and was calculated using the following formula: $1.96\times \sqrt{SE_{1}^{2}+SE_{2}^{2}}$. Whereby $SE_{1}$ is the individual’s IRT estimated SE of the T-score at baseline and $SE_{2}$ at the 2-week measurement. A mean MDC of each domain was subsequently calculated for the whole group.

### Responsiveness

Responsiveness (defined as the ability of a PROM to detect change over time in the construct to be measured)^19^ of the PROMIS CATs was determined by comparing changes in PROMIS CAT T-scores to changes in the GRS (online supplemental table 3).^33^ On average, we had no expectations on the change scores, as the population included is diverse (ranging from recently diagnosed, being well regulated for T2D, having T2D and CKD). Also, 6 months is a relatively short period of time for a chronic disease such as T2D. Therefore, we expected relatively low correlations. However, we expected that there would be at least some variation in outcomes and that some participants would improve and some participants would deteriorate and that this variation would be reflected in the change scores on the PROMIS CATs.^34^ To support the responsiveness of PROMIS CAT, we hypothesised that the correlations between changes in PROMIS CAT T-scores and changes in comparable domains of the comparator instruments would be at least 0.40 (rather than 0.50 suggested by COSMIN), comparable to the paper of Terwee *et al*.^15^ We examined the ability of the PROMIS CATs to distinguish between patients who reported to be deteriorated (a little deteriorated or much deteriorated on the GRS) and patients who reported to be not deteriorated (not changed, a little improved, much improved on the GRS). The area under the receiver operating characteristics (ROC) curve (AUC) was used as a measure of responsiveness. An AUC of at least 0.70 is generally considered sufficient evidence for responsiveness.^30^ We considered responsiveness sufficient if at least 75% of the results were in accordance with the hypotheses. Since there was little change in scores, we decided not to assess minimal important change (MIC), defined as the smallest change that participants consider important.^35^ Only participants with complete baseline and follow-up measurements per domain were included in the analysis.

#### Stage 2: acceptability

All focus groups and interviews were audio-recorded and transcribed verbatim by two researchers and one research assistant, and all personal identifying information was removed from the transcripts. Any ambiguities in the transcripts were listened to again and clarified where necessary. The transcripts were analysed using the Framework approach.^36^ Two independent researchers coded the transcripts based on the topic lists. Subsequently, the coded transcripts were arranged to broader themes. Differences were discussed until consensus was reached. Qualitative data were analysed using Excel. As an indication of the relative importance of the themes, we produced a bar graph, indicating the frequency of coding this theme in the transcripts (quotation frequency).

## Results

### Stage 1: assessment of validity, reliability and responsiveness

#### Study participants

The baseline questionnaires were completed by 314 people with T2D (age 64.0±10.8 years, 63.7% male, average diabetes duration 5.7±4.3 years, and mean HbA1c level was 53.6±11.2 mmol/mol and 7.0±1.0%), of which 282 completed the 2 weeks and 279 completed the 6-month measurement (table 1).

**Table 1:** Baseline characteristics of 314 people with T2D

Demographic variables	
Age (years)	64.0 (10.8)
Men (%)	63.7
Low educational level (%) (n=150)	31.3
Western ethnicity (%) (n=220)	92.3
Diabetes duration (years) (n=186)	5.7 (4.3)
Smoking, current and former (%) (n=241)	69.3
Metabolic variables	
Haemoglobin A1c (HbA1c, mmol/mol and %) (n=253)	53.6 (11.2) and 7.0 (1.0)
Fasting glucose (mmol/l) (n=195)	8.8 (2.2)
Total cholesterol (mmol/l) (n=248)	4.4 (0.9)
LDL cholesterol (mmol/l) (n=248)	2.3 (0.8)
HDL cholesterol (mmol/l) (n=248)	1.2 (0.3)
Triglycerides (mmol/l) (n=248)	1.7 (1.2–2.4)
BMI (kg/m^2^) (n=246)	31.2 (5.1)
Systolic/diastolic blood pressure (mm Hg) (n=199)	138.1 (17.9)/81.1 (8.5)
Kidney function, eGFR (n=264)	85.0 (24.0)
Psychosocial variables	
SF-12 score	
Physical component summary*	42.2 (10.9)
Mental component summary*	50.0 (10.2)
ADDQoL score (n=247)	
Overall QoL score	
In general, my present QoL is … (% good, very good or excellent)	78.5
If I did not have diabetes, my quality of life would be … (% a little better, much better or very much better)	51.8
Weighted impact score Leisure Activities domain	−1.1 (0.1)
PHQ-9 score (n=247)	3 (1–7)
Depressive symptoms (PHQ-9≥10) (%)	15.3
EuroQol five dimensions (EQ-5D) score (n=247)	
Mobility (% no problems in walking about)	59.5
Self-care (% no problems with self-care)	96
Usual activities (% no problems with performing usual activities)	66
Pain/discomfort (% no pain or discomfort)	43.3
Anxiety/depression (% not anxious or depressed)	84.6
PAID20 score (n=247)	5 (1.3–16.3)
Severe diabetes distress (PAID20≥40) (%)	11.3
PROMIS Global Health Scale score (n=249)	
Global Physical Health	45.5 (9.9)
Global Mental Health	48.5 (9.2)
PROMIS scores	
v1.0 PROMIS Pain Intensity (0–10)	2 (0–5)
v1.2 PROMIS Physical Functioning	46.1 (8.2)
v1.1 PROMIS Pain Interference	52.2 (9.1)
v1.0 PROMIS Fatigue	50.6 (9.2)
v1.0 PROMIS Sleep Disturbance	49.0 (8.2)
v1.0 PROMIS Anxiety	50.7 (7.9)
v1.0 PROMIS Depression	48.6 (7.7)
v2.0 PROMIS Ability to Participate in Social Roles and Activities	51.1 (8.1)

Percentages, mean (SD) or median (IQR).

* SF-12 physical component summary includes the domains physical functioning, role-physical, bodily pain and general health; SF-12 mental component summary includes the domains vitality, social functioning, role-emotional and mental health.

ADDQoL, audit of diabetes dependent quality of life; BMI, body mass index; eGFR, estimated glomerular filtration rate; HDL, high-density lipoprotein; HRQOL, health-related quality of life; LDL, low-density lipoprotein; PAID20, Problem Areas in Diabetes Scale 20; PHQ-9, Patient Health Questionnaire-9; PROMIS, Patient-Reported Outcomes Measurement Information System; QOL, quality of life; SF-12, Short-Form Health Survey 12; T2D, type 2 diabetes.

#### Construct validity

We observed sufficient construct validity for six out of the seven PROMIS CATs (table 2). For Sleep Disturbances, 32 out of 33 hypotheses were confirmed; for Pain interference, 29 out of 33 hypotheses; for Physical Function and Depression, 27 out of 33 hypotheses; for Anxiety, 26 out of 33 hypotheses; and for Ability to participate in social roles and activities, 25 out of 33 hypotheses were met. Only Fatigue did not meet the requirements for sufficient construct validity, since only 68% of the results were in agreement with the hypotheses (22 out of 33 hypotheses were confirmed).

**Table 2:** Pearson’s correlations for construct validity for seven PROMIS CATs in people with T2D

	PROMIS Physical Functioning	PROMIS Pain Interference	PROMIS Fatigue	PROMIS Sleep Disturbance	PROMIS Anxiety	PROMIS Depression	PROMIS Ability to Participate
SF-12 Physical functioning	**0.72**	*−0.65*	−0.59	−0.35	−0.43	−0.39	0.51
SF-12 Role physical	**0.72**	−0.64	−0.67	−0.39	−0.48	−0.46	**0.60**
SF-12 Bodily pain	*0.71*	**−0.84**	−0.64	−0.37	−0.47	−0.41	0.55
SF-12 General health	*0.63*	−0.52	−0.67	−0.43	−0.49	−0.42	0.50
SF-12 Vitality	0.60	−0.53	**−0.74**	−0.41	−0.43	−0.42	0.51
SF-12 Social functioning	0.58	−0.51	−0.62	−0.43	−0.52	−0.55	**0.65**
SF-12 Role emotional	0.37	−0.38	−0.45	−0.41	−0.55	−0.60	**0.45**
SF-12 Mental health	0.33	−0.34	−0.46	−0.43	**−0.65**	**−0.73**	0.43
SF-12 PCS	**0.80**	*−0.75*	−0.68	−0.35	−0.40	−0.31	0.56
SF-12 MCS	0.29	−0.28	−0.49	−0.45	**−0.60**	**−0.68**	0.47
EQ-5D Mobility	**−0.65**	0.54	0.41	0.22	0.29	0.30	−0.40
EQ-5D Self-care	**−0.21**	0.29	0.23	0.22	0.19	0.20	−0.26
EQ-5D Usual activities	**−0.61**	0.53	0.69	0.37	0.47	0.49	−0.58
EQ-5D Pain/discomfort	−0.53	**0.70**	0.54	0.32	0.40	0.39	−0.41
EQ-5D Anxiety/depression	−0.17	0.23	0.30	0.37	**0.51**	**−0.34**	0.07
PHQ-9	−0.48	0.49	0.70	**0.54**	0.61	**0.60**	−0.54
PAID20	−0.16	0.19	0.39	0.21	**0.34**	**0.34**	−0.27
ADDQoL general QoL	−0.58	0.49	0.61	0.40	0.57	0.63	−0.63
ADDQoL impact diabetes on QoL	0.13	−0.15	−0.28	−0.12	−0.18	−0.15	0.18
ADDQoL Leisure activities	0.18	−0.13	−0.26	−0.12	−0.14	−0.13	**0.21**
Pain intensity	−0.52	**0.76**	0.49	0.34	0.36	0.37	−0.37
PROMIS Global01	−0.63	0.55	0.68	0.44	0.49	0.40	−0.46
PROMIS Global02	−0.53	0.44	0.53	0.39	0.49	0.52	−0.49
PROMIS Global03	**−0.61**	0.48	0.60	0.37	0.45	0.38	−0.47
PROMIS Global04	−0.34	0.36	0.45	0.38	**0.58**	**0.63**	−0.43
PROMIS Global05	−0.46	0.39	0.50	0.40	0.50	0.51	**−0.55**
PROMIS Global09r	−0.48	0.38	0.51	0.34	0.41	0.45	**−0.55**
PROMIS Global06	**−0.73**	0.65	0.62	0.33	0.39	0.34	−0.55
PROMIS Global10r	−0.32	0.34	0.40	0.47	**0.72**	**0.69**	−0.45
PROMIS Global08r	−0.55	0.51	**0.80**	0.43	0.45	0.44	−0.47
PROMIS Global07r	−0.57	**0.77**	0.50	0.32	0.39	0.34	−0.40
GPH	**0.73**	*−0.71*	*−0.75*	−0.44	−0.50	−0.46	0.57
GMH	0.49	−0.46	−0.55	−0.48	**−0.68**	**−0.71**	**0.58**
Hypotheses confirmed (%)	82	88	67	97	79	82	76

Correlations in bold were expected to be strong (≥0.7 or ≤−0.7), correlations in italic were expected to be moderate (0.5–0.7), other correlations were expected not to be strong (≤0.0 or ≥−0.6).

ADDQoL, audit of diabetes dependent quality of life; CAT, computerised adaptive test; EQ-5D, EuroQol five dimensions; GMH, global mental health; GPH, global physical health; MCS, mental component summary score; PAID20, Problem Areas in Diabetes Scale 20; PCS, physical component summary score; PHQ-9, Patient Health Questionnaire-9; PROMIS, Patient-Reported Outcomes Measurement Information System; QoL, quality of life; SF-12, Short-Form Health Survey 12; T2D, type 2 diabetes.

#### Test–retest reliability

All seven PROMIS CAT domains showed sufficient test–retest reliability, with ICCs between 0.73 (for Ability to Participate in Social Roles and Activities) and 0.91 (for Physical Function). For the PROMIS Pain Intensity single item, the ICC was 0.72 (table 3).

**Table 3:** Reliability measures, including ICC agreement, SEM and MDC of the seven PROMIS CATs and PROMIS Pain Intensity in people with T2D

Tool and domain	ICC agreement (95% CI)	SEM	MDC
PROMIS CAT			
Physical Function	0.91 (0.89 to 0.93)	2.05	5.69
Pain Interference	0.85 (0.81 to 0.88)	2.66	7.42
Fatigue	0.84 (0.80 to 0.87)	2.07	5.74
Sleep Disturbance	0.84 (0.80 to 0.87)	2.24	6.21
Anxiety	0.79 (0.74 to 0.83)	2.29	6.36
Depression	0.82 (0.77 to 0.85)	2.40	6.69
Ability to Participate in Social Roles and Activities	0.73 (0.67 to 0.78)	2.09	5.82
PROMIS single item			
Pain Intensity (0–10)	0.72 (0.66 to 0.78)	1.51	4.17

CAT, computerised adaptive test; ICC, intraclass correlation coefficient; MDC, minimal detectable change; PROMIS, Patient-Related Outcomes Measurement Information System; SEM, standard error of measurement; T2D, type 2 diabetes.

The SEM and MDC of the PROMIS CAT domains ranged from 2.05 to 2.66, and from 5.69 and 7.42 T-score points, respectively (table 3). Small MDCs can distinguish small changes from measurement errors with 95% CI. For example, an MDC of 6 points means that when an individual patient changes 6 points, we are 95% sure that the patient has really changed (ie, that this change is not due to measurement error).

#### Responsiveness

For all PROMIS CAT domains, the correlations (as depicted in table 4) were lower than expected, generally ranging from 0.09 to 0.31. Because the correlations between the PROMIS CAT change scores and the GRS were too low for all domains, except for Fatigue, an MIC was not calculated. The areas under the ROC curve for each domain are depicted in table 5. All PROMIS CAT domains showed almost no changes in scores, which resulted in insufficient responsiveness, as only for PROMIS CAT Fatigue and Depression, 50% of the results were in accordance with the hypotheses, which was less than 75%. For the other PROMIS CATs, none of the results were in accordance with the hypotheses.

**Table 4:** Expected and observed correlations for responsiveness between PROMIS CAT change scores and changes in Global Rating Scale scores

	PROMIS CAT domains						
	Physical Function	Pain Interference	Fatigue	Sleep Disturbance	Anxiety	Depression	Ability to Participate
PROMIS single item							
Pain Intensity (0–10)	−0.26	0.42	0.27	0.26	0.13	0.12	−0.17
Global Rating Scale							
Change in Physical Function	**0.21** *	−0.25	−0.27	−0.07	−0.15	−0.16	0.21
Change in Pain Interference	−0.18	**0.29** *	0.18	0.16	0.1	0.02	−0.18
Change in Fatigue	−0.24	0.23	**0.31** *	0.06	0.19	0.19	−0.22
Change in Sleep Disturbance	−0.09	0.19	0.18	**0.25** *	0.12	0.25	−0.09
Change in Anxiety	−0.05	0.08	0.18	0.12	**0.09** *	0.22	0.01
Change in Depression	−0.03	0.07	0.16	0.08	0.08	**0.20** *	−0.01
Change in Ability to Participate	0.15	−0.14	−0.19	−0.1	−0.11	−0.09	**0.16** *

Bold=The PROMIS CAT was expected to have the highest correlations with the Global Rating scales measuring similar domains.

* Expected correlations of at least 0.40.

CAT, computerised adaptive test; PROMIS, Patient-Reported Outcomes Measurement Information System.

**Table 5:** Area under the ROC curve (AUC), representing the ability of PROMIS CATs to distinguish patients who deteriorated from patients who did not deteriorate

	AUC
PROMIS CAT	
Physical Function	0.59
Pain Interference	0.69
Fatigue	0.71
Sleep Disturbance	0.66
Anxiety	0.67
Depression	0.74
Ability to Participate in Social Roles and Activities	0.49

CAT, computerised adaptive testing; PROMIS, Patient-Reported Outcomes Measurement Information System; ROC, receiver operating characteristic.

### Stage 2: acceptability

In total, five people with T2D participated in focus groups that took place on 25 October 2021 and 26 October 2021. Their mean age was 69.0 years (±8.8 years) and 60% were men.

In the focus groups, most participants agreed that all seven PROMIS domains (Depression, Sleep Disturbances, Fatigue, Anxiety, Pain Interference, Physical Functioning and Ability to Participate in Social Roles and Activities) are relevant (online supplemental figure 1), since they feel the majority of the domains are related. For example, one of the participants commented: ‘I think most domains are important and related; in my own case, sleep disturbance is caused by pain, and this results in fatigue.’ Second, when asked which domain is most important, Physical Function appeared to be most relevant, followed by Pain Interference, Sleep Disturbance and Fatigue, then Anxiety and Depression and finally Ability to Participate in Social Roles and Activities. The latter was least important (but still relevant) and a participant indicated that ‘it is not diabetes in itself that prevents me from participating in social activities, but rather the consequences of diabetes, which sometimes make it difficult to engage in conversation’. Third, when asked if the participants missed any topics or domains, two topics came up: comorbidities and medication use, since participants felt the need to clarify that many of these domains were not (only) related to, or influenced by, their diabetes. Finally, with regard to the acceptability of CATs in care, they felt that completing CAT questionnaires prior to visiting the doctor could be an efficient way to start the conversation between patient and their doctor as well as provide the patient with more confidence. The participants all felt, however, that ‘questionnaires should never replace personal consultations with the physician’. If implemented in regular care, participants would be willing to spend approximately 15 min to complete CATs.

In total, nine healthcare professionals (66.7% women, their mean age was 50.7 years), including general practitioners, specialist diabetes nurses, physiotherapists, psychologists, a psychotherapist specialised in diabetes and a professor specialised in PROMs and CATs, participated in the interviews between 18 January and 5 May 2022. In the interviews, participants expressed that in general, they feel it is important to use PROMIS CATs in diabetes care in order to know how a patient is doing, both physically and mentally. One of the healthcare providers said: ‘We all know that it is very important to know how someone is doing, apart from all the medical data and lab results’. Unlike the participants in the focus groups, healthcare providers found the domains Anxiety and Depression to be important and they indicated that they currently miss questions on lifestyle, such as eating behaviour and physical activity. In addition, they indicated that time and knowledge are currently the most important barriers for implementing CATs in clinical practice. In their opinion, there should be enough time prior to the consultation to explain to people with diabetes why completing the CATs is important, and enough time during the consultations to discuss the topics that need more attention. Furthermore, they feel that a proper introduction of the CATs to people with diabetes is crucial, indicating that: ‘An introduction is very important. To indicate that there is no right or wrong answer. People should not have the idea that something depends on these questionnaires. Or that they will be labelled. It’s not a diagnosis. Tell them that it is more about creating awareness and making it something that can be discussed. It is important to make that clear’. Regarding the feedback of results, they said that a colour system would be preferred, so that the healthcare provider can clearly see which topics need extra attention, which makes interpretation of the results easier than when using scores. They too feel that approximately 15 min should be acceptable for patients to complete the CATs.

## Discussion

In our study, we observed that in 314 people with T2D, 6 out of 7 PROMIS CAT domains demonstrated evidence for sufficient construct validity. Only the PROMIS Fatigue CAT did not show sufficient construct validity. All seven PROMIS CATs showed sufficient test–retest reliability and none showed sufficient responsiveness. During the focus groups and interviews, people with T2D and healthcare providers agreed that CATs could serve as a conversation starter in routine care, but they should never replace personal consultations with the doctor.

We are the first to examine the validity, reliability, responsiveness and acceptability of PROMIS CATs in people with T2D. With regard to construct validity, our results were very comparable to results in patients with advanced CKD,^7 15^ chronic back pain,^37^ tendon rupture^38^ and lumbar spinal stenosis.^39^ We showed that all domains had sufficient validity except for Fatigue, which showed sufficient construct validity in patients with CKD,^7 15^ but not in our T2D population (≥75%). Positive results were the high correlations of PROMIS Fatigue CAT with the SF-12 subscale Vitality and the fatigue item of PROMIS Global Health (Global08r). However, correlations with other SF-12 and EQ-5D domains, such as physical functioning/usual activities and general health were higher than expected. This may be due to the content of the PROMIS Fatigue item bank, which does not only include items measuring fatigue severity, but also fatigue impact (eg, ‘To what degree did your fatigue interfere with your physical functioning?’). However, some correlations were inconsistent (eg, a high correlation with PHQ-9 (measuring depression) and a low correlation with EQ-5D item anxiety/depression), which is more difficult to explain.

Test–retest reliability, measured as ICC agreement, SEM and MDC, showed similar results compared with the assessments in patients with CKD^7 15^ and lumbar spinal stenosis.^39^ In the studies on patients with chronic back pain^37^ and tendon rupture,^38^ no assessment of the CATs test–retest reliability was made. In people with T2D, all CAT domains showed higher ICCs (better reliability) and small MDCs (low measurement error) compared with the other included PROMs. Small MDCs are desirable as they allow for small changes to be distinguished from measurement error with 95% confidence in individual patients. Overall, in people with T2D, the CATs in all domains showed sufficient test–retest reliability.

With regard to responsiveness, in contrast to studies in chronic back pain,^37^ tendon rupture^38^ or lumbar spinal stenosis,^39^ which assessed changes after treatment (operations or interventions), we observed small changes in PROMIS CAT scores. Our results were similar to the study in advanced CKD patients,^7 15^ which also observed small changes in PROMIS CAT scores. The small changes could be due to the relatively short follow-up periods during which most patients did not change (ranging from 60.3% for Physical Function to 85.7% for Anxiety), which is in line with a recent other publication.^40^ This is an important limitation of the design of this study, because a requirement for assessing responsiveness is that at least part of the participants change.^41^ This also resulted in too low correlations with the GRS to estimate MIC values.

Longer follow-up studies are, therefore, needed to assess responsiveness of PROMIS CATs in people with T2D. Based on previous studies on fatigue in the same cohort, a follow-up period between at least 1 and 7 years would be suitable in order to detect sufficient changes in these domains.^42 43^ Other studies, however, provide indirect evidence for responsiveness of PROMIS PROMs in people with T2D. For example, PROMIS sleep measures were more likely than other questionnaires to detect an improvement with positive airway pressure therapy in people with T2D and obstructive sleep apnoea.^44^ This indirect evidence does not guarantee responsiveness of the CATs, as responsiveness can be distorted by floor or ceiling effects. However, due to the large item banks that feed into the CATs, floor and ceiling effects are seldom found for PROMIS CAT.^45^ Furthermore, the studies in patients with acute disorders undergoing treatment who experienced larger changes in PROM scores, including chronic back pain,^37^ tendon rupture^38^ or lumbar spinal stenosis^39^ did show good responsiveness of the PROMIS CATs.

The results regarding the acceptability showed PROMIS CATs to be acceptable for use in clinical care by both healthcare providers and people with T2D. We studied the acceptability of the CATs using only a few focus groups and interviews, with also a limited number of participants. Therefore, large-scale implementation research on using PROMIS CATs in routine care for people with T2D is still needed. A recent large-scale implementation study in an outpatient orthopaedics clinic tested the implementation of three PROMIS CATs (Physical Function, Pain Interference, Depression) in orthopaedic patients.^46^ The PROMIS CATs were filled out by over 20.000 patients. The average time to complete was less than 5 min, while registration times for new patients did not change significantly, showing successful implementation to collect patient-reported outcomes using CATs and directly import the results into the electronic medical record in real time for use during the clinical visit.^46^

This study has some limitations that need to be discussed. One limitation of PROMIS CATs in general is that CATs can only be completed digitally. Participants thus have to have access to an electronic device and be digitally skilled. In the Netherlands, approximately 80% of the population aged 55 years or older is sufficiently digitally skilled,^47^ but in many other countries, citizens are less digitally skilled.^48^ Consequently, it may be challenging to reach the entire T2D population. In our study, we enabled participation by telephone, but none were willing to participate in this manner. For routine care, other methods could be considered, such as offering help or making tablets available on site. Another practical issue is that the use of software is necessary to link the electronic patient file to CAT software. However, there are already commercial parties supplying this service (ie, KLIK, https://www.hetklikt.nu). Additionally, in contrast to short forms, using CATs is associated with costs. A third limitation is that we used a high precision stopping rule, which resulted in a relatively large number of more than 40 questions asked in total (median: 47, IQR: 40–58), which is still less than when completing regular PROMs. A total of 40 questions means that on average, 6 questions per domain were asked, where measuring depression through the Centre for Epidemiologic Studies Depression Scale or measuring sleep disturbances through the General Sleep Disturbance Scale (GSDS), for instance, would take 20 or 21 items, respectively.^49 50^ If fewer items are preferred, alternative stopping rules can be used. For example, the standard stopping rule will result in 36–43 items in total (5–6 items per CAT), with a minimum of 29 items since the standard stopping rule requires 4 items per CAT. However, the standard stopping rule of PROMIS, with a precision of 0.9 and maximum of 8–12 items per CAT, may result in lower precision and lower responsiveness. A fourth limitation during the acceptability research is that we used the names of the PROMIS domains, which may have influenced participants. For example, asking participants if they feel depressed or anxious may scare them off and they might answer no, while when using less official language, for example, asking them if they worry about the future, this may feel more relatable and they may answer yes. This might also explain why the Anxiety and Depression domains were barely mentioned during the focus groups, while healthcare providers indicated them as important domains for people with diabetes. Another limitation of the acceptability part of this study is that we cannot strongly conclude that data saturation has been reached, since only two focus groups with a total of only five people with T2D could be scheduled. However, the most important issues were mentioned.

Using PROMs in T2D care can support the delivery of person-centred care. In the Netherlands, PROMIS has been selected as the preferred measurement system for use in care, across all disciplines.^51^ Administering seven PROMIS CATs (and in the future perhaps even more, as more PROMIS CATs have been developed and translated that are relevant for people with T2D, such as Cognitive Function and Sexual Function) takes more time than completing one general PROM, such as the SF-12. However, seven PROMIS CATs can be administered in an average of 10 min, which people with T2D have indicated they find acceptable and will provide much more detailed information. Already present are ways to provide graphical feedback on CATs, which improves interpretability by the healthcare provider, facilitates conversations with patients^52^ and are available in some electronic health records (eg, Epic). We, therefore, recommend using PROMIS CATs in clinical practice.

## Conclusions

PROMIS CATs showed sufficient construct validity and test–retest reliability in most domains (Physical Function, Pain Interference, Sleep Disturbance, Anxiety, Depression and Ability to Participate in Social Roles and Activities) in people with T2D. Additionally, the PROMIS CATs showed insufficient responsiveness, because most participants’ own rating of their health compared with 6 months ago did not change. Therefore, responsiveness needs to be evaluated in a population with poorer diabetes control or in a study design with longer follow-up. The CATs are well accepted to be used in care to identify relevant topics, but should not replace personal contact with the doctor.

## Supplementary material

10.1136/bmjopen-2024-087898 online supplemental file 1

10.1136/bmjopen-2024-087898 online supplemental table 1

10.1136/bmjopen-2024-087898 online supplemental table 2

10.1136/bmjopen-2024-087898 online supplemental table 3

## Data Availability

Data are available on reasonable request.
