# Supplementary material for: Assessment of validity, reliability, responsiveness and acceptability of seven Dutch-Flemish PROMIS computerised adaptive tests (CATs) in Dutch people with type 2 diabetes: an observational and qualitative study
Source: BMJ Open. 2025 Nov 28;15(11):e087898. doi: 10.1136/bmjopen-2024-087898 (PMC12684131; doi:10.1136/bmjopen-2024-087898)

Supplementary Figure 1. Bar graph indicating the relevance of each of the seven PROMIS CAT domains, their acceptability, and other relevant topics that were discussed during the focus groups

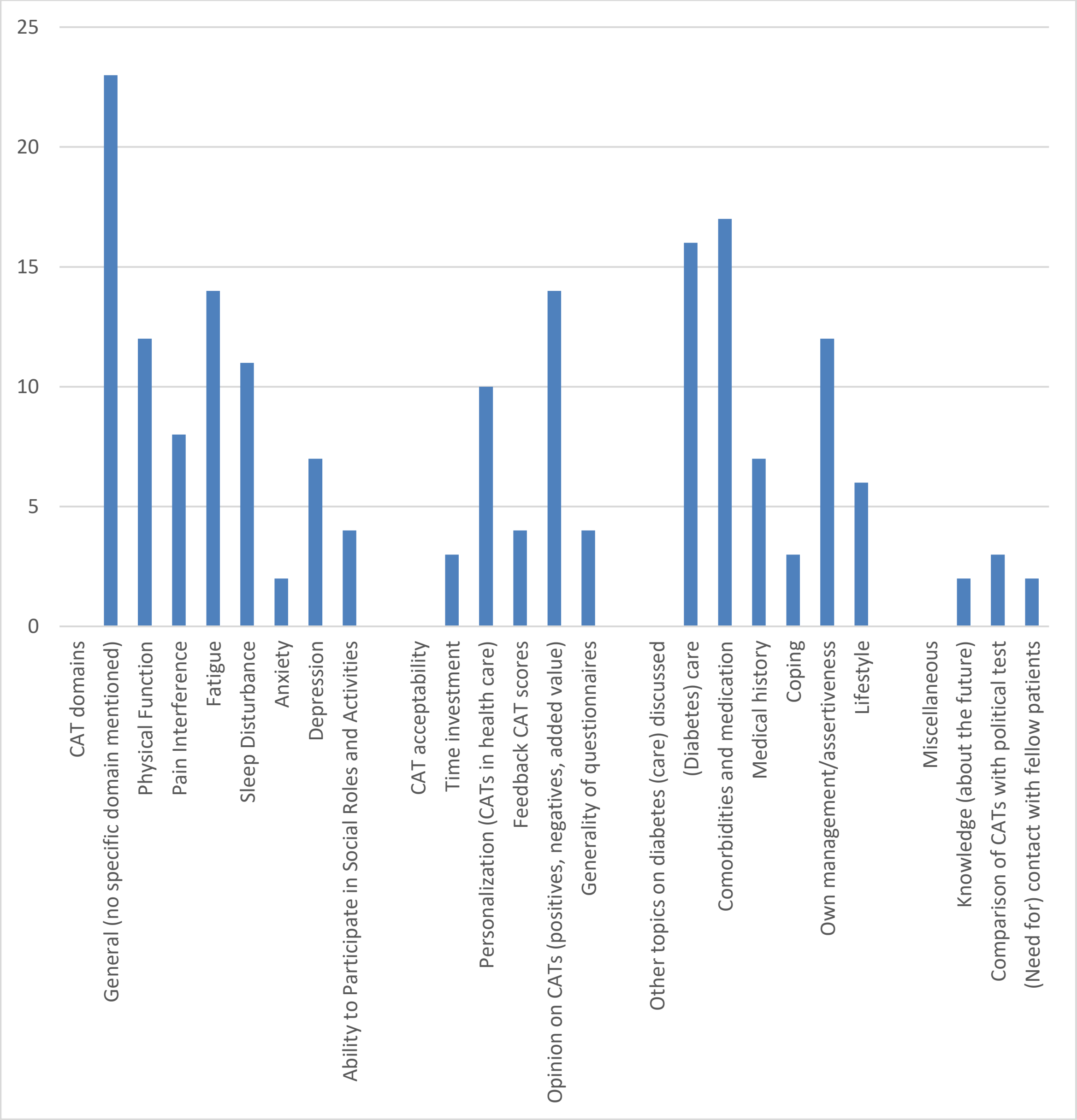

Supplement: online supplemental file 1 [file bmjopen-15-11-s001.pdf]
