# Supplementary material for: Assessment of validity, reliability, responsiveness and acceptability of seven Dutch-Flemish PROMIS computerised adaptive tests (CATs) in Dutch people with type 2 diabetes: an observational and qualitative study
Source: BMJ Open. 2025 Nov 28;15(11):e087898. doi: 10.1136/bmjopen-2024-087898 (PMC12684131; doi:10.1136/bmjopen-2024-087898)
Supplement: online supplemental table 1 [file bmjopen-15-11-s002.docx]

**Supplementary Table 1. Scripts for the focus group and interview**

***Script for the focus group with people with type 2 diabetes***

--- Recently, questionnaires to measure patient reported outcomes on various aspects of self-rated

health have been developed. Before these questionnaires can be used in daily practice

or research, we first must examine whether the domains that these questionnaires cover are

relevant. The purpose of this meeting is to investigate whether you think the selection of

questionnaires is complete or if you miss domains or if some domains are irrelevant for the

measurement of self-rated health. In response to the following questions, we ask you to

discuss and debate your answer with each the other participants. In the meantime, the

discussion leader will keep an eye on the equal input of every participant. There are

questionnaires in seven domains, namely Anxiety, Depression, Fatigue, Pain Interference,

Physical Function, Sleep Disturbance, Ability to Perform Social Roles and Activities as well as Satisfaction with Social Roles and Activities.

1) What do you think of these domains (first impression)?

2) Are these domains the most relevant to assess the impact of diabetes? And could you

rate them, one being most important, seven least important?

3) How important/urgent is it to measure patient related outcomes per respective domain

in regular care for people with diabetes?

4) Today, we wanted to gain insight into all the domains of self-rated health in which

diabetes has an impact. Have I forgotten to ask you something that relates to this

subject? Is there something you like to say if you did not have the opportunity during the

discussion? Would you like to add something to the previous discussions? ---

The following questions are asked during the second part of the focus group:

---- ‘Welcome back after the break, in the second part of the focus group, we will discuss how to implement the previously discussed questionnaires in clinical practice and how we should feedback their results to doctors and people with T2D. In response to the following questions, we ask you to discuss and debate your answer with each other. In the meantime, the discussion leader will keep an eye on the equal input of every participant.

1) Potential: Why would you like to have PROs measured? How useful will measurement of PROs be? Which goals (personal or practice) would measurement of PROs fit? What should the goal of measuring PROs through these questionnaires be?

2) Capability: What are currently barriers for measuring measure PROs in regular care for people with diabetes? Is it i.e. time, access to questionnaires, security issues, sensitive subject, difficult subject? What are the factors in current practice that facilitate measuring patient related outcomes in regular care for people with diabetes? Could this be achieved by i.e. Tablet-PCs or laptops, extra time consultation?

3) Capacity: Do you think it is your responsibility to share your PROs as a person with diabetes? What is needed of fellow people with T2D to implement PROs?

4) Contribution: Does the proposed pilot make sense to you, what aspects do you like or not? How much time are you willing to put into this pilot? Do you think the pilot will be beneficiary for you?

5) Summary: today, we wanted to gain insight into how we can implement the validated CATs in clinical practice and feedback their results to doctors and people with T2D. Have I forgotten to ask you something that relates to this subject? Is there something you like to say if you did not have the opportunity during the discussion? Would you like to add something to the previous discussions? ----

***Script for the interview with healthcare providers***

- To begin with, could you tell me what your experience in general has been in administering the PROMIS CATs? What comes to mind first when you think of CATs?
- What effects did you, as a department/research group, want to achieve with the CATs?
- Before I ask about the patient’s perspective, I would like to hear your thoughts on various aspects of administering CATS.

Is there a need for training/education, mutual sharing of experiences, e.g. how do I discuss the results with patients?

- What are barriers to administering/completing CATs?
- Administering 🡪 healthcare providers
- Completing 🡪 patients
- What are facilitators for administering/completing CATs?
- Administering 🡪 healthcare providers
- Completing 🡪 patients
- Is there a need for guidance in completing the CATs?
- Is it common for people to start but not complete the CATs? Do you know that the reason for this is (e.g. is it due to login problems or is it something else)? Does this occur more often in certain groups, for example the elderly who are less computer savvy with online questionnaires?
- What is your experience in discussing the PROMIS CATs (e.g. is it clear to patients and healthcare providers what each result means? Does discussing the results take long, is it easy?)
- Which form of feedback appeals most to patients? For example, do they prefer pictures, colors, etc.? Do they appreciate the comparison of their own results with other patients or not?
- How should we implement CATs relevant to people with T2D in regular care?
- What are success factors and what are the barriers to implementing CATs?
- What time investment and costs are required for implementation?
- Time investment patients 🡪 How much time are your patients willing to spend on completing CATs?
- Time investment healthcare providers 🡪 How much time do they have in their daily care provision to attend CATs training? How much time do they have to explain CATs to patients? How much time do GPs/specialists have to discuss outcomes, etc.?
- Which CATs should at least remain implemented?
- Is there a need for adjustments?
