# Supplementary material for: Assessment of validity, reliability, responsiveness and acceptability of seven Dutch-Flemish PROMIS computerised adaptive tests (CATs) in Dutch people with type 2 diabetes: an observational and qualitative study
Source: BMJ Open. 2025 Nov 28;15(11):e087898. doi: 10.1136/bmjopen-2024-087898 (PMC12684131; doi:10.1136/bmjopen-2024-087898)
Supplement: online supplemental table 2 [file bmjopen-15-11-s003.docx]

| PROMIS CAT | Strong correlation  Pearson’s r ≥ 0.7 | Moderate correlation  Pearson’s r 0.5-0.7 | No strong correlation  Pearson’s r ≤ 0.6 |
| --- | --- | --- | --- |
| Physical functioning | SF-12 Physical functioning  SF-12 Role Physical  SF-12 Physical component summary  EQ-5D Mobility  EQ-5D Self-care  EQ-5D Usual activities  PROMIS Global health Global03  PROMIS Global health Global06  Global Physical Health | SF-12 General health  SF-12 Bodily pain | All other domains |
| Pain interference | SF-12 Bodily pain  EQ-5d Pain/discomfort  PROMIS Global health Global07r  Pain intensity | SF-12 Physical functioning  SF-12 Physical component summary  Global Physical Health | All other domains |
| Fatigue | PROMIS Global health Global08r  SF-12 Vitality | Global Physical Health | All other domains |
| Sleep disturbance | PHQ-9 |  | All other domains |
| Anxiety | EQ-5D Anxiety/depression  PROMIS Global Health Global04  PROMIS Global Health Global10r  Global Mental Health  PAID20  SF-12 Mental Health  SF-12 Mental component summary |  | All other domains |
| Depression | PHQ-9  EQ-5D Anxiety/depression  PROMIS Global Health Global04  PROMIS Global Health Global10r  Global Mental Health  PAID20  SF-12 Mental Health  SF-12 Mental component summary |  | All other domains |
| Ability to participate in social roles and activities | ADDQoL Leisure activities  PROMIS Global Health Global05  PROMIS Global Health Global09r  Global Mental Health  SF-12 Social Functioning  SF-12 Role Physical  SF-12 Role Emotional |  | All other domains |

**Supplementary Table 2. Hypotheses for the expected correlations for construct validity of seven PROMIS CATs in people with T2D**
