# Supplementary material for: Assessment of validity, reliability, responsiveness and acceptability of seven Dutch-Flemish PROMIS computerised adaptive tests (CATs) in Dutch people with type 2 diabetes: an observational and qualitative study
Source: BMJ Open. 2025 Nov 28;15(11):e087898. doi: 10.1136/bmjopen-2024-087898 (PMC12684131; doi:10.1136/bmjopen-2024-087898)
Supplement: online supplemental table 3 [file bmjopen-15-11-s004.docx]

**Supplementary Table 3. Mean (SD) change in PROMIS CAT T-scores across levels of self-rated change in the same construct**

|  | Physical function | | Pain interference | | Fatigue | | Sleep disturbance | | | Anxiety | | | Depression | | Ability to participate | |
| --- | --- | --- | --- | --- | --- | --- | --- | --- | --- | --- | --- | --- | --- | --- | --- | --- |
|  | N (%) | Mean (SD) change | N (%) | Mean (SD) change | N (%) | Mean (SD) change | N (%) | Mean (SD) change | N (%) | | Mean (SD) change | N (%) | | Mean (SD) change | N (%) | Mean (SD) change |
| Much improved | 18 (6.6) | 1.2 (4.1) | 56 (20.7) | -3.3 (7.0) | 16 (5.9) | -3.0 (5.3) | 10 (3.7) | -1.6 (4.4) | 13 (4.8) | | -2.6 (4.9) | 18 (6.7) | | -2.5 (6.7) | 8 (3.0) | 2.8 (4.2) |
| A little improved | 33 (12.1) | -0.1 (3.6) | 36 (13.3) | -1.1 (7.1) | 27 (10.0) | -0.8 (8.3) | 20 (7.4) | -2.4 (3.6) | 20 (7.4) | | 0.4 (5.0) | 22 (8.2) | | -2.4 (4.6) | 29 (10.8) | -1.7 (6.5) |
| Not changed | 164 (60.3) | -0.2 (4.0) | 126 (46.5) | -0.1 (5.8) | 152 (56.3) | -0.1 (5.3) | 205 (75.9) | 0.2 (5.2) | 231 (85.6) | | -0.1 (5.6) | 219 (81.4) | | -0.2 (5.1 | 201 (75.0) | 0.5 (6.1) |
| A little worse | 49 (18.0) | -1.7 (4.9) | 50 (18.5) | 2.5 (5.2) | 65 (24.1) | 2.9 (5.3) | 29 (10.7) | 2.1 (4.8) | 5 (1.9) | | 2.6 (6.9) | 9 (3.3) | | 4.2 (5.2) | 23 (8.6) | -2.7 (6.1) |
| Much worse | 8 (2.9) | -4.3 (3.3) | 3 (1.1) | 3.1 (2.5) | 10 (3.7) | 6.9 (4.7) | 6 (2.2) | 7.3 (10.4) | 1 (0.4) | | 0.00 | 1 (0.4) | | 3.6 | 7 (2.6) | -8.3 (6.8) |
|  |  |  |  |  |  |  |  |  |  | |  |  | |  |  |  |
| Total improved | 51 (18.7) |  | 92 (34.0) |  | 43 (15.9) |  | 31 (11.1) |  | 33 (12.2) | |  | 41 (14.9) | |  | 37 (13.8) |  |
| Total deteriorated | 57 (20.9) |  | 53 (19.6) |  | 75 (27.8) |  | 35 (12.9) |  | 6 (2.3) | |  | 10 (3.7) | |  | 30 (11.2) |  |
