# Supplementary material for: Assessment of validity, reliability, responsiveness and acceptability of seven Dutch-Flemish PROMIS computerised adaptive tests (CATs) in Dutch people with type 2 diabetes: an observational and qualitative study
Source: BMJ Open. 2025 Nov 28;15(11):e087898. doi: 10.1136/bmjopen-2024-087898 (PMC12684131; doi:10.1136/bmjopen-2024-087898)
Supplement: Supplementary file 5 [file bmjopen-2024-087898.reviewer_comments.pdf]

PEER REVIEW HISTORY

BMJ Open publishes all reviews undertaken for accepted manuscripts. Reviewers are asked to complete a checklist review form and are provided with free text boxes to elaborate on their assessment. These free text comments are reproduced below.

ARTICLE DETAILS

Title (Provisional)

Assessment of validity, reliability, responsiveness and acceptability of seven Dutch-Flemish PROMIS computerized adaptive tests (CATs) in Dutch people with type 2 diabetes: an observational and qualitative study

Authors

Groeneveld, Lenka; Langendoen-Gort, Marlous; Pals, Fleur; Blom, Marieke T; Terwee, Caroline; van der Willik, Esmee M.; van Ittersum, Frans J.; Beulens, Joline; Elders, Petra; Rutters, Femke

---

VERSION 1 - REVIEW

**Reviewer**	**1**
**Name**	**Fenwick, Eva**
**Affiliation**	**Singapore Eye Research Institute**
**Date**	**06-Aug-2024**
**COI**	**None**

This paper reports on the validity of seven Dutch-Flemish PROMIS CATs in adults with type 2 diabetes (T2D) using various psychometric assessments, and evaluates their acceptability using qualitative methods. The paper is well-written and the study appears overall well designed and carried out. However, there are several clarifications required before the paper is suitable for publication (attached in file).

Psychometric properties and acceptability of seven Dutch-Flemish PROMIS computerized adaptive tests (CATs) in Dutch people with type 2 diabetes

Overall

This paper reports on the validity of seven Dutch-Flemish PROMIS CATs in adults with type 2 diabetes (T2D) using various psychometric assessments, and evaluates their acceptability using qualitative methods. The paper is well-written and the study appears overall well designed and carried out. However, there are several clarifications required before the paper is suitable for publication.

Major comments

1. Some references to back up the claims made about the limitations of current DM-related QoL tools is needed in the first paragraph of the introduction – has a review been conducted on the measurement properties of DM-related HRQOL PROMs?

2. It also seems strange to lay out a knowledge gap that a consensus is needed on which aspects of HRQOL should be measured in T2DM yet not explicitly address this in the paper. The authors explore which PROMIS CAT domains are considered relevant to patients and providers but this isn't the same thing as finding out which domains should be measured in the first place. Moreover, the use of generic HRQOL PROMs rather than DM-specific PROMs for DM patients is questionable, as patients with DM may have specific issues not captured in the PROMIS CATs. Indeed, in the qualitative portion, it is reported that patients felt DM-specific issues like medication use were not covered. There is no justification of why the PROMIS generical HRQOL CATs were chosen for this study.
3. The wording in the abstract and main text methods seems misleading as it suggests that test-retest reliability was evaluated with ICC *and* SEM *and* MDC, whereas the latter two tests appear to be measuring psychometric properties separate to test-retest? If not, I struggle to see how SEM and MDC are indicators of test-retest reliability…
4. The responsiveness assessment appears strange given there was no intervention so it's not surprising that the scores did not change over time. Traditionally, isn't responsiveness of an instrument referring to its ability to detect change in a person's scores pre/post some intervention? Not by correlating the CAT scores with a global outcome question. The reference to Terwee et al [12] isn't correct so I could not evaluate what the authors were trying to achieve with this effort. Is there a reference that states that responsiveness can be evaluated without an intervention? Their conclusion that the PROMIS CATs were not responsive may need to be tempered with some more detail – e.g. "not responsive to patients' own rating of their health compared to 6 months ago using a single recall item." Otherwise this could mislead readers who just read the abstract that the PROMIS CATs are not response to DM treatments/interventions. Indeed, in the discussion, the authors acknowledge that their results are different to those evaluating responsiveness following treatment/intervention. Overall, this section of the study does not add much value and is distracting to readers, and the authors should consider removing it.
5. It would have been informative to also show useability outcomes, such as no. items needed for each CAT, SE obtained for each CAT score (test precision), time taken for each CAT, etc.
6. There is a disconnect between what the qualitative work is stated to measure ("Acceptability" of actual usage) and what was reported (Scope of use, willingness to use by patients, and relevance of PROMIS domains for patients). It would be good to use an accepted framework/definition for assessing Acceptability (e.g. Proctor's outcomes in implementation science). It is also not clear when the qualitative portion was completed – had the patients participating in the qualitative interviews already completed the PROMIS CATS as part of the normal assessment (i.e. had they had actual experience in completing the CATs)? Or were they naïve to the CATs and the researchers provided a description of how they worked? Please provide a couple of example questions from the interview guide in the main text.
7. A limitation of the study is that patients were excluded if they didn't have access to an e-device – this is likely to have skewed acceptability findings as those without tech available were not interviewed. The authors acknowledge this as a limitation of the CATs themselves, but it should be acknowledged more specifically as a limitation of the study design.
8. The focus groups must have been very small if only 5 people participated – how many in each group and how many groups? Usually the minimum for a viable focus group is ~3 otherwise it's more like a semi-structured interview. Moreover, this sample size was small, even for qualitative work – how much confidence can be put in these results? Was thematic saturation reached?
9. Were there any data collected on response rate? E.g. what proportion of those who were invited to participate answered the CATs? What proportion dropped out over the 3 time periods? This would be a good quantitative measure of acceptability. Also, it would be interesting to look at uptake (response) rate by demographic variables, e.g. were there any differences in participants who did and didn't answer the baseline CATs – this could show issues with acceptability in certain groups. Similarly was there an association between sociodemographic variables and drop out rate over time?

Minor comments

1. In the abstract, there is a lack of information in the results. Need to give some more data e.g. what does 'sufficient construct validity' mean, how is this quantified? What was the cut-off in correlations to assume responsiveness was 'insufficient'.
2. Some editing for English-language fluency would benefit the paper.

3. A sample size of 100 was deemed to be sufficient according to COSMIN guidelines – but what assessment was this for specifically in the guidelines, as there are a lot of psychometric assessments conducted in this study and they may have different sample size requirements.
4. Lines 11-15 on page 8 are not a complete sentence.
5. It seems odd to include the ADDQOL then only use 3 items from it – if a general QOL question was needed for convergent validity testing, perhaps a generic HRQOL should have been used. Moreover, the ADDQOL's complicated multiplicated scoring system has known psychometric issues and any scores computed from this questionnaire should be interpreted with caution.[1]
6. Please give some information about the HCPs e.g. age, gender, years in profession
7. Line 33 page 16 – what does 'they' refer to in the sentence "…and they indicated that they currently miss questions on lifestyle…" Does 'they' refer to the HCPs or the CATs?
8. One of the limitations of the study is the heavy testing protocol at baseline (perhaps too many PROMs?) that may have contributed to drop outs at the 2 week and 6 month timeframe. What implications does the drop out rate have on the generalizability of the conclusions drawn?
9. The title of Supplementary Fig 1 is simply 'Bar graph' – a more informative title and caption explaining the results is needed.
10. Ref 12 is provided to show that the current study was designed according to COSMIN guidelines for ?psychometric analyses of PROMs? but the actual ref 12 appears to be for guidelines for systematic reviews of PROMs?

References

1. Finger RP, Fenwick E, Pesudovs K, Marella M, Lamoureux EL, Holz FG. Rasch analysis reveals problems with multiplicative scoring in the macular disease quality of life questionnaire. *Ophthalmology*. Nov 2012;119(11):2351-7. doi:10.1016/j.ophtha.2012.05.031

**Reviewer**	**2**
**Name**	**Kerari, Ali**
**Affiliation**	**King Saud University College of Nursing**
**Date**	**07-Oct-2024**
**COI**	**I do understand the above.**

Dear Authors,

Many thanks for the opportunity to review the manuscript: "Psychometric properties and acceptability of seven Dutch- Flemish PROMIS computerized adaptive tests (CATs) in Dutch people with type 2 diabetes." The manuscript has the potential to contribute to the understanding of how clinicians can use the validated PROMIS CATs among Dutch individuals with T2D. I have the following notes for your consideration:

Abstract

- Considering the issue in the introduction and method, the abstract must be changed accordingly.

Introduction

- While the manuscript has strengths, such as evaluating the construct validity of PROMIS CATs, the scale and its subscales are not clearly explained and can be confusing at times.

The authors can compare and contrast between the PROMS (no CATs) and the of PROMIS CATs.

- Before delving into the aim of your study, please include information on the use of PROMIS CATs in healthcare settings, as well as any evaluations of the psychometric properties of this scale in other languages.

Methods

- The authors stated "The study was designed and reported according to COSMIN guidelines." Provide a brief explanation of COSMIN guidelines to enhance readership.
- Page 8, Line 8-11: This paragraph is a bit confusing. Please provide succinct information on each subscale, if applicable.
- The authors sated "we used the following regular PROMs (not CATs) to assess construct validity of the seven PROMIS CATs." I believe that this statement was not adequately addressed in the aim of your study. Please revised.
- Construct Validity: The authors used Pearson's correlations to assess construct validity of the PROMIS CATs. The authors can use specific terms related to the evaluation of construct validity, such as convergent validity (assessing correlations between similar constructs) and divergent validity (assessing correlations between different constructs).

Discussion and Study Implication

- The discussion reflects the problems in the introduction and the method. It would benefit from better structure.
- Page 17/Line 47-52: Were there any inconsistent correlations reported in other similar studies?

Quality of English Language

I am not qualified to assess the quality of English in this paper

Thank you and good luck.

VERSION 1 - AUTHOR RESPONSE

Reviewer 1:

Dear Authors, Many thanks for the opportunity to review the manuscript: "Psychometric properties and acceptability of seven Dutch- Flemish PROMIS computerized adaptive tests (CATs) in Dutch people with type 2 diabetes." The manuscript has the potential to contribute to the understanding of how clinicians can use the validated PROMIS CATs among Dutch individuals with T2D. I have the following notes for your consideration:

Abstract

• Considering the issue in the introduction and method, the abstract must be changed accordingly.

We changed the abstract according to the comments of the editor.

Introduction

• While the manuscript has strengths, such as evaluating the construct validity of PROMIS CATs, the scale and its subscales are not clearly explained and can be confusing at times. The authors can compare and contrast between the PROMS (no CATs) and the of PROMIS CATs.

Thank you for pointing out this confusion. We have added information to the introduction, hoping that it's clearer now. The added information now reads: "The nationally and internationally recommended Patient-Reported Outcomes Measurement Information System (PROMIS®) is a collection of PROMs, used to measure a variety of HRQOL domains [5, 6] [7]. These PROMs capture several domains that are commonly relevant across diseases. For each domain, different types of PROMs were developed. The basis consists of an item bank for each domain, containing a large number of questions. From this item bank short forms of four to eight items were developed. In addition, the item bank can be used for computerized adaptive testing (CAT), an efficient administration method in which the computer selects questions from an item bank based on the answers to previous questions, until a predefined reliability or a maximum number of items is administered. With CATs, PROMs are adapted to the symptom severity or functional level of the patient, generally resulting in less items and more relevant questions [8]. The psychometric properties as well as feasibility of using PROMIS CATs have recently been evaluated in other populations, such as rehabilitation inpatients [9], people with stroke [10] and people with advanced chronic kidney disease [11]. PROMIS CATs have shown to have better measurement properties than traditional generic PROMs and similar ones as disease-specific PROMs, making them suitable for use in clinical practice [12-14]."

• Before delving into the aim of your study, please include information on the use of PROMIS CATs in healthcare settings, as well as any evaluations of the psychometric properties of this scale in other languages.

Thank you for your suggestion. We added this information to the introduction which now reads: "The psychometric properties as well as feasibility of using PROMIS CATs have recently been evaluated in other populations, such as rehabilitation inpatients [9], people with stroke [10] and people with advanced chronic kidney disease [11]. According to the Dutch-Flemish PROMIS initiative, PROMIS CATs have better measurement properties than traditional generic PROMs and similar ones as disease-specific PROMs, making them suitable for use in clinical practice [12]."

Methods

• The authors stated "The study was designed and reported according to COSMIN guidelines." Provide a brief explanation of COSMIN guidelines to enhance readership.

We added this information, which now reads:"The study was designed and reported according to COSMIN guidelines. In short, in 2021 a literature review and Delphi rounds were used to produce a COSMIN guideline for studies on measurement properties of PROMs. Consensus was achieved on a set of 71 items for inclusion of reporting guideline for studies on measurement properties of PROMs, which will guide researchers on the necessary information to report on."

• Page 8, Line 8-11: This paragraph is a bit confusing. Please provide succinct information on each subscale, if applicable.

Thank you for pointing out any confusion. We rephrased this section, which now reads:" The impact question asks how participants would enjoy their leisure activities if they would not have diabetes. This question consists of five response options, ranging from -3 ('a lot more') to 1 ('less'). The importance question asks how important leisure activities are to the participants and consists of four response options, ranging from 0 ('very important') to 3 ('not at all important')."

• The authors sated "we used the following regular PROMs (not CATs) to assess construct validity of the seven PROMIS CATs." I believe that this statement was not adequately addressed in the aim of your study. Please revised.

We added this information to the introduction and adjusted the aim, which now reads:" This study aimed to assess construct validity against commonly used PROMs, test-retest reliability and responsiveness of the above mentioned seven Dutch-Flemish PROMIS CATs in Dutch adults with T2D, and assess their acceptability in health care providers and people with T2D."

• Construct Validity: The authors used Pearson's correlations to assess construct validity of the PROMIS CATs. The authors can use specific terms related to the evaluation of construct validity, such as convergent validity (assessing correlations between similar constructs) and divergent validity (assessing correlations between different constructs).

Thank you for this suggestion. We added this information to the 'Construct validity' section under '(Statistical) analyses'. Those sentences now read: "Regarding convergent validity, we hypothesized strong correlations ($r \geq 0.7$) between PROMIS CATs and comparable PROM domains and moderate correlations ($r=0.5 – 0.7$) between PROMIS CATs and largely related PROM domains. Regarding divergent validity we hypothesized weaker correlations ($r \leq 0.6$) for other comparisons (Supplementary Table 2)."

Discussion and Study Implication
• The discussion reflects the problems in the introduction and the method. It would benefit from better structure.

We changed the discussion according to the change in the introduction.

• Page 17/Line 47-52: Were there any inconsistent correlations reported in other similar studies?

Other validation studies assessing the same CATs report similar correlations. For example, Van der Willik et al. assessed the correlations between the same seven PROMIS CATs against the SF-12 in a population of people with chronic kidney disease (also referred to in the manuscript) and found correlations in the same directions. Some of the correlations were a little weaker than the ones we found, but no differences worth mentioning were found.

Quality of English Language
I am not qualified to assess the quality of English in this paper
Thank you and good luck

Thank you.

Reviewer 2:
Psychometric properties and acceptability of seven Dutch-Flemish PROMIS computerized adaptive tests (CATs) in Dutch people with type 2 diabetes

Overall
This paper reports on the validity of seven Dutch-Flemish PROMIS CATs in adults with type 2 diabetes (T2D) using various psychometric assessments, and evaluates their acceptability using qualitative methods. The paper is well-written and the study appears overall well designed and carried out. However, there are several clarifications required before the paper is suitable for publication.

Thank you.

Major comments

1. Some references to back up the claims made about the limitations of current DM-related QoL tools is needed in the first paragraph of the introduction – has a review been conducted on the measurement properties of DM-related HRQOL PROMs?

We agree and elaborated on this in the introduction, which now reads:"In our systematic review on patient-reported outcome measures (PROMs) for people with type 2 diabetes (T2D), we showed that a large number of PROMs is available, which intend to measure (aspects of) health-related quality of life (HRQOL) [1-4]. However, the PROMs currently used in the field of T2D have limitations: the PROMs measure a large variety of (sub)constructs, which are not all HRQOL constructs, with a small number of PROMs not measuring HRQOL at all. There is a need for consensus on which aspects of HRQOL should be measured in people with T2D and which PROMs to use in research and daily practice."

2. It also seems strange to lay out a knowledge gap that a consensus is needed on which aspects of HRQOL should be measured in T2DM yet not explicitly address this in the paper. The authors explore which PROMIS CAT domains are considered relevant to patients and providers but this isn't the same thing as finding out which domains should be measured in the first place. Moreover, the use of generic HRQOL PROMs rather than DM-specific PROMs for DM patients is questionable, as patients with DM may have specific issues not captured in the PROMIS CATs. Indeed, in the qualitative portion, it is reported that patients felt DM-specific issues like medication use were not covered. There is no justification of why the PROMIS generical HRQOL CATs were chosen for this study.

We agree and elaborated on this in the introduction, which now reads:" The psychometric properties as well as feasibility of using PROMIS CATs have recently been evaluated in other populations, such as rehabilitation inpatients [9], people with stroke [10] and people with advanced chronic kidney disease [11]. PROMIS CATs have shown to have better measurement properties than traditional generic PROMs and similar ones as disease-specific PROMs, making them suitable for use in clinical practice [12-14].
Seven of those Dutch-Flemish PROMIS CATs, which assess several general HRQoL domains, are: 1) Physical Function, 2) Pain Interference, 3) Fatigue, 4) Sleep Disturbance, 5) Anxiety, 6) Depression, and 7) Ability to Participate in Social Roles and Activities. At the moment no T2D specific domains, such as diabetes distress are available yet as CATs. The domains that are available showed sufficient construct validity against commonly used PROMs and test-retest reliability in Dutch patients with advanced Chronic Kidney Disease (CKD) [11]. However, we do not know the psychometric properties of these CATs in Dutch people with T2D and whether and how they could be implemented in routine care. This study aimed to assess construct validity against commonly used PROMs, test-retest reliability and responsiveness of the above mentioned seven Dutch-Flemish PROMIS CATs in Dutch adults with T2D, and assess their acceptability in health care providers and people with T2D."

3. The wording in the abstract and main text methods seems misleading as it suggests that test-retest reliability was evaluated with ICC and SEM and MDC, whereas the latter two tests appear to be measuring psychometric properties separate to test-retest? If not, I struggle to see how SEM and MDC are indicators of test-retest reliability…

According to the international COSMIN taxonomy, reliability includes two measurement properties: (1) reliability (the proportion of the total variance in the measurements which is due to true differences between patients) and (2) measurement error (the systematic and random error of a patient's score that is not attributed to true changes in the construct to be measured). We have now made this distinction in the abstract and included these definitions in the methods section.

The section now reads: “Two aspects of test-retest reliability were assessed: reliability (defined as the proportion of the total variance in the measurements which is due to true differences between patients) and measurement error (defined as the systematic and random error of a patient’s score that is not attributed to true changes in the construct to be measured)[18].
To assess reliability, the intraclass correlation coefficient (ICC) was calculated in participants with a valid baseline and 2-weeks measurement. A measurement is considered valid when both measurements were completed by the participants within a maximum of 28 days. The ICC was calculated using a two-way random-effects model for absolute agreement: $ICC\ agreement = \frac{\sigma_p^2}{\sigma_p^2+\sigma_m^2+\sigma_e^2}$,
whereby σ_p^2 is the variation between patients, σ_m^2 is the variation between measurements and σ_e^2 is the random error variance. The ICC was computed for each CAT domain as well as PROMIS Pain Intensity single item separately. We considered an ICC ≥ 0.70 as sufficient.

To assess measurement error, the standard error of measurement (SEM) was calculated for each CAT domain separately. SEM was calculated by dividing the standard deviation of the difference between test and retest scores (SDdiff) by √2. A mean SEM of each domain was subsequently calculated for the whole group. The SEM is expressed in the unit of measurement of the PROM scale and lower scores mean less measurement error.

The minimal detectable change (MDC) was calculated to facilitate interpretation of PROMIS change scores in clinical practice. The MDC was calculated for each CAT domain separately. The MDC is defined as the smallest change in score that can be detected beyond measurement error, with 95% confidence. Being based on item response theory (IRT), PROMIS CATs, the MDC differs per participant (because with IRT the standard error (SE) of each score is different) and was calculated using the following formula: $1.96 * \sqrt{SE_1^2 + SE_2^2}$. Whereby SE_1 is the individual’s IRT estimated standard error of the T-score at baseline and SE_2 at the 2-weeks measurement. A mean MDC of each domain was subsequently calculated for the whole group.

4. The responsiveness assessment appears strange given there was no intervention so it’s not surprising that the scores did not change over time. Traditionally, isn’t responsiveness of an instrument referring to its ability to detect change in a person’s scores pre/post some intervention? Not by correlating the CAT scores with a global outcome question. The reference to Terwee et al [12] isn’t correct so I could not evaluate what the authors were trying to achieve with this effort. Is there a reference that states that responsiveness can be evaluated without an intervention? Their conclusion that the PROMIS CATs were not responsive may need to be tempered with some more detail – e.g. “not responsive to patients’ own rating of their health compared to 6 months ago using a single recall item.” Otherwise this could mislead readers who just read the abstract that the PROMIS CATs are not response to DM treatments/interventions. Indeed, in the discussion, the authors acknowledge that their results are different to those evaluating responsiveness following treatment/intervention. Overall, this section of the study does not add much value and is distracting to readers, and the authors should consider removing it.

Responsiveness refers to the ability of a PROM to detect change over time in the construct to be measured. This does not necessarily have to be a change in a person’s scores pre/post some intervention. Changes can, for example, also happen because of the natural course of a disease. The general assumption to be tested in a responsiveness study is that if a person changes in the construct of interest, this change should be reflected in the change score on the PROM. To assess whether a person has changed, an external criterion is needed. For this purpose, often a global rating scale is used. We added a reference that explains this design.

We agree that we could not show sufficient responsiveness of the PROMIS CATs using this follow-up duration in this current population, which was more stable than expected, but that in future studies it may be possible to demonstrate responsiveness using another design (e.g. before/after an intervention) or a different population with poorer glycemic control. We modified the results section and conclusion in the abstract, now stating that "Responsiveness was rated as insufficient in this study design as there was almost no change in participants' own rating of their health compared to six months ago according to a global rating of change." and "Responsiveness needs to be evaluated in a population with poorer diabetes control or in a study design with longer follow-up." Additionally, we prefer to keep that section in the discussion paragraph, because it reflects the limitation of our study design and provides an explanation why other studies found other results.

5. It would have been informative to also show useability outcomes, such as no. items needed for each CAT, SE obtained for each CAT score (test precision), time taken for each CAT, etc.

The usability outcomes were described in the method section, which reads: "In this study, the CAT stopped when a standard error (SE) of 2.2 on the T-score metric was reached (comparable to a reliability of approximately 0.95) or when a maximum of 12 items per CAT was administered." Unfortunately, time taken to complete each CAT was not recorded.

6. There is a disconnect between what the qualitative work is stated to measure ("Acceptability" of actual usage) and what was reported (Scope of use, willingness to use by patients, and relevance of PROMIS domains for patients). It would be good to use an accepted framework/definition for assessing Acceptability (e.g. Proctor's outcomes in implementation science). It is also not clear when the qualitative portion was completed – had the patients participating in the qualitative interviews already completed the PROMIS CATS as part of the normal assessment (i.e. had they had actual experience in completing the CATs)? Or were they naïve to the CATs and the researchers provided a description of how they worked? Please provide a couple of example questions from the interview guide in the main text.

Thank you for these questions. Regarding the "Acceptability" part of your question we have added information on the used framework. This section now reads: "In the second part of the focus group, using the principles of the Extended Normalization Process Theory (ENPT), we discussed how to implement the CATs in clinical practice and how we should feedback their results to doctors and people with T2D, by asking "What are the factors in current practice that facilitate measuring patient related outcomes in regular care for people with diabetes? Could this be achieved by i.e. Tablet-PCs or laptops, extra time consultation?". ENPT conceptualises the socio-structural and social-cognitive resources that people draw on to realise an implementation and offers a useful framework for explaining implementation processes and the role played by intervention context interactions. ENPT has four main constructs, namely potential (concerns, change valence and change efficacy), capability (workability and integration), capacity (structure, resources, norms and roles) and contribution (for which potential, capability and capacity form the context). The discussion was focused on what the participants identified as chances and options but also risk and limitations.".

Regarding the second part of your question, we have added information on when the focus groups took place with respect to completing the CATs. Additionally, we have added a few example questions from the supplemental file on the focus group and interview script in the main text. The 'Stage 2: acceptability' part under Measures from the Method section, now reads: "The focus groups took place at the end of October 2021, after participants had completed the baseline, two-weeks and six-months measurements. Each focus group and interview took about one hour, was

audio recorded and transcribed verbatim. During the focus groups and interviews a standardized script including predefined questions was followed. For the full list of questions, please see Supplementary Table 1. In short, first the different PROMIS domains were introduced and it was then assessed whether the domains were considered relevant, for example by asking the participants: “Are these domains the most relevant to assess the impact of diabetes? And could you rate them, one being most important, seven least important?”. Furthermore, we assessed whether the whole set was considered comprehensive for the measurement of self-rated health. In the second part of the focus group, we discussed how to implement the CATs in clinical practice and how we should feedback their results to doctors and people with T2D, by asking “What are the factors in current practice that facilitate measuring patient related outcomes in regular care for people with diabetes? Could this be achieved by i.e. Tablet-PCs or laptops, extra time consultation?”.

7. A limitation of the study is that patients were excluded if they didn’t have access to an e-device – this is likely to have skewed acceptability findings as those without tech available were not interviewed. The authors acknowledge this as a limitation of the CATs themselves, but it should be acknowledged more specifically as a limitation of the study design.

Participants without access to an electronic device with internet connection were offered the option to participate over the phone. However, they were not willing to participate in this way. We elaborated on this in the discussion and added it to the limitation section after the abstract. This now reads: “A second limitation is that, although offered an alternative way to participate, participants that did not have access to an electronic device with internet connection, did not participate.” and “In our study, we enabled participation by telephone, but none were willing to participate in this manner.”

8. The focus groups must have been very small if only 5 people participated – how many in each group and how many groups? Usually the minimum for a viable focus group is ~3 otherwise it’s more like a semi-structured interview. Moreover, this sample size was small, even for qualitative work – how much confidence can be put in these results? Was thematic saturation reached?

We agree that the focus groups were very small and we had hoped to include more participants, but unfortunately, due to the focus groups being scheduled during office hours and them being held online due to COVID-19, it was very hard to find available participants. Therefore, we cannot strongly conclude that data saturation has been reached and have added this limitation to the Discussion section.

9. Were there any data collected on response rate? E.g. what proportion of those who were invited to participate answered the CATs? What proportion dropped out over the 3 time periods? This would be a good quantitative measure of acceptability. Also, it would be interesting to look at uptake (response) rate by demographic variables, e.g. were there any differences in participants who did and didn’t answer the baseline CATs – this could show issues with acceptability in certain groups. Similarly was there an association between sociodemographic variables and drop out rate over time?

Thank you for these questions. We have added a flowchart to provide the response rate and drop out. Exact numbers on the participants with CKD and T2D, who were recruited in Amsterdam, were reported elsewhere (https://doi.org/10.1093/ndt/gfac231). Please note that the numbers reported in that paper also include participants without T2D who were not included in our current analyses.

Minor comments

1. In the abstract, there is a lack of information in the results. Need to give some more data e.g. what does ‘sufficient construct validity’ mean, how is this quantified? What was the cut-off in correlations to assume responsiveness was ‘insufficient’.

We added this information to the Results section of the abstract, which now reads: ”Except for Fatigue, all PROMIS CAT domains demonstrated sufficient construct validity, since ≥75% of the results was in accordance with a priori hypotheses. All seven PROMIS CATs showed sufficient test-retest reliability (ICCs 0.73-0.91) and SEM and MDC ranged from 2.1 to 2.7 and from 5.7 to 7.4, respectively. Responsiveness was rated as insufficient in this study design as there was almost no change in participants’ own rating of their health compared to six months ago using a single recall item”.

2. Some editing for English-language fluency would benefit the paper.

Thank you for your suggestion. As none of the co-authors is a native English speaker we did as best as we could. Based on your comment, we asked AI for suggestions to improve English-language fluency in the Introduction and Discussion.

3. A sample size of 100 was deemed to be sufficient according to COSMIN guidelines – but what assessment was this for specifically in the guidelines, as there are a lot of psychometric assessments conducted in this study and they may have different sample size requirements.

According to the COSMIN study design checklist that can be found on the COSMIN website, this applies to construct validity, test-retest reliability and responsiveness. A sample size of at least 100 even takes into account the expected number of missing values. We added this information to the Methods section, which now reads: “According to COSMIN, a sample size of 100 was considered “very good” for construct validity, test-retest reliability and responsiveness, so we more than complied with these guidelines”.

4. Lines 11-15 on page 8 are not a complete sentence.

We have checked our original submission and cannot find the incomplete sentence. In our document these lines on page 8 read: “A weighted impact score can then be calculated by multiplying the impact rating and importance rating, ranging from -9 (maximum negative impact of diabetes) to 3 (maximum positive impact of diabetes). Higher scores indicate better quality of life.”

5. It seems odd to include the ADDQOL then only use 3 items from it – if a general QOL question was needed for convergent validity testing, perhaps a generic HRQOL should have been used. Moreover, the ADDQOL’s complicated multiplicated scoring system has known psychometric issues and any scores computed from this questionnaire should be interpreted with caution.[1]

We agree with this comment and in hindsight would have chosen a different approach, but unfortunately, there’s nothing we can do about this now.

6. Please give some information about the HCPs e.g. age, gender, years in profession

We added details that were known to the Results part discussing the acceptability, which now reads: ”In total, nine health care professionals (66.7% women, age 50.7 years), including general practitioners, specialist diabetes nurses, physiotherapists, psychologists, a psychotherapist specialized in diabetes and a professor specialized in PROMs and CATs, participated in the interviews between January 18th and May 5th 2022.”

7. Line 33 page 16 – what does 'they' refer to in the sentence "…and they indicated that they currently miss questions on lifestyle…" Does 'they' refer to the HCPs or the CATs?

'They' refers to the health care providers.

8. One of the limitations of the study is the heavy testing protocol at baseline (perhaps too many PROMs?) that may have contributed to drop outs at the 2 week and 6 month timeframe. What implications does the drop out rate have on the generalizability of the conclusions drawn?

Thank you for this interesting question. We agree that perhaps many regular PROMs had to be completed at baseline and that this might have attributed to some of the drop outs. However, when the study procedures and measurements were explained over the phone at inclusion, we have informed participants that the baseline measurement would be the longest, and that more questionnaires had to be completed, compared to the two weeks and six months measurements. Another plausible explanation for participants dropping out when this study was executed, is the COVID-19 pandemic, as a few study participants pointed out to be (mentally) occupied during that time.

Additionally, we don't think that a drop out of 32 participants between baseline and two weeks, and another 3 participants between two weeks and six months (drop out of 11.1% in total) would have had any major impact on the generalizability of our conclusions.

9. The title of Supplementary Fig 1 is simply 'Bar graph' – a more informative title and caption explaining the results is needed.

Thank you for pointing this out. We have changed the title to "Bar graph indicating the relevance of each of the seven PROMIS CAT domains, their acceptability, and other relevant topics that were discussed during the focus groups".

10. Ref 12 is provided to show that the current study was designed according to COSMIN guidelines for ?psychometric analyses of PROMs? but the actual ref 12 appears to be for guidelines for systematic reviews of PROMs?

We added another reference that discusses the COSMIN guidelines.

References
1. Finger RP, Fenwick E, Pesudovs K, Marella M, Lamoureux EL, Holz FG. Rasch analysis reveals problems with multiplicative scoring in the macular disease quality of life questionnaire. Ophthalmology. Nov 2012;119(11):2351-7. doi:10.1016/j.ophtha.2012.05.031

VERSION 2 - REVIEW

**Reviewer**	**2**
**Name**	**Kerari, Ali**
**Affiliation**	**King Saud University College of Nursing**

Date **05-May-2025**

COI

Dear Authors,

Thank you for your interesting paper. I have the following notes for your consideration:

Title

- Please change “psychometric properties” to “validation.” The authors did not use exploratory or confirmatory factor analysis methods to evaluate the psychometric properties of the scale. Therefore, I suggest using the term “validation” instead of “psychometric properties.”

Introduction

- This first paragraph should be placed at the end of the introduction section. The authors should begin with two to three paragraphs explaining the background or the use of the seven Dutch-Flemish PROMIS in the context of chronic diseases.
- PROMs have been validated in the context of chronic diseases, such as chronic kidney disease and stroke. I do not think this will add new information if the authors testing the scale’s validation among patients with diabetes. Any justifications**?**

Methods

- Authors stated that “A total of 840 potential participants were contacted of which 372 were interested in receiving the patient information letter. After receiving the letter, 315 participants provided informed consent”. Calculating the appropriate sample size for a validation study involves several factors, including the number of items. Which specific guidelines did the authors use to calculate the sample size for the study?
- Regarding divergent validity, we hypothesized weaker correlations ($r \leq 0.6$) for other comparisons.” It is a bit confusing. To assess divergent validity, measures should not be related or they are indeed not correlated or have weak correlations. Any justifications?

Results

- The result section was well-written.

Discussion

- “However, some correlations were inconsistent (e.g. a high correlation with PHQ-9 (measuring depression) and a low correlation with EQ-5D item anxiety/depression), which is more difficult to explain.” I believe the authors followed a specific statistical method to evaluate convergent and divergent validity, which may have produced these results. Considering alternative statistical methods could potentially yield significant findings.

Quality of English Language

I am not qualified to assess the quality of English in this paper.

Thank you and good luck.

VERSION 2 - AUTHOR RESPONSE

Reviewer: 2

Dear Authors,

Thank you for your interesting paper. I have the following notes for your consideration:

Title

• Please change “psychometric properties” to “validation.” The authors did not use exploratory or confirmatory factor analysis methods to evaluate the psychometric properties of the scale. Therefore, I suggest using the term “validation” instead of “psychometric properties.”

Thank you for this comment. We agree and changed it to validity, reliability and responsiveness throughout the paper.

Introduction

• This first paragraph should be placed at the end of the introduction section. The authors should begin with two to three paragraphs explaining the background or the use of the seven DutchFlemish PROMIS in the context of chronic diseases.

Thank you for this comment. We agree and changed the order of the introduction.

• PROMs have been validated in the context of chronic diseases, such as chronic kidney disease and stroke. I do not think this will add new information if the authors testing the scale’s validation among patients with diabetes. Any justifications?

Thank you for this question. We agree that PROMs have been validated in other populations already. However, not in people with type 2 diabetes. We suspected that in our population other topics, such as sexual function would play an important role too and therefore wanted to investigate whether the PROMIS CATs used in this study would cover all relevant topics. We added this justification to the introduction.

Methods

• Authors stated that “A total of 840 potential participants were contacted of which 372 were interested in receiving the patient information letter. After receiving the letter, 315 participants provided informed consent”. Calculating the appropriate sample size for a validation study involves several factors, including the number of items. Which specific guidelines did the authors use to calculate the sample size for the study?

Based on COSMIN guidelines 100 participants should suffice and adding another 100 would add a little more precision. As we wanted to account for participants dropping out of the study, especially during challenging COVID times, and since participating was not suspected to be too burdensome, we decided to aim for 300 inclusions.

• Regarding divergent validity, we hypothesized weaker correlations ($r \leq 0.6$) for other comparisons.” It is a bit confusing. To assess divergent validity, measures should not be related or they are indeed not correlated or have weak correlations. Any justifications?

We used the terms convergent and divergent validity to make a distinction between expected strong correlations ($r \geq 0.7$) between PROMIS CATs and comparable PROM domains and weaker correlations ($r \leq 0.6$) between PROMIS CATs and other domains. We expected that some of these correlations would not be so low, because some domains are related (e.g. anxiety and depression) even though they are considered different constructs. The cut-off between convergent and divergent validity is arbitrary.

Results
• The result section was well-written.

Thank you.

Discussion
• "However, some correlations were inconsistent (e.g. a high correlation with PHQ-9 (measuring depression) and a low correlation with EQ-5D item anxiety/depression), which is more difficult to explain." I believe the authors followed a specific statistical method to evaluate convergent and divergent validity, which may have produced these results. Considering alternative statistical methods could potentially yield significant findings.

We used correlation analyses to evaluate convergent and divergent validity, which is a common approach for assessing validity. Because a lot of correlations were calculated there is always a possibility to find some unexpected results. Therefore, we used the criterion that 75% of the results should be in accordance with the hypotheses. Alternative methods could have been assessing known groups validity, but it is often difficult to hypothesize expected differences in scores between relevant subgroups. We don't think this would have led to a different conclusion.

Quality of English Language
I am not qualified to assess the quality of English in this paper.

Thank you and good luck

VERSION 3 - REVIEW

**Reviewer**	**2**
**Name**	**Kerari, Ali**
**Affiliation**	**King Saud University College of Nursing**
**Date**	**28-Aug-2025**
**COI**	

I have no further comments.

Good luck.
